# Accurate and Sensitive Multiplexed Proteomics Using the 7‑Plex DiLeuC Complementary Ion Strategy

**DOI:** 10.1021/jasms.6c00234

**Published:** 2026-10-06

**Authors:** Jingwei Zhang, Zicong Wang, Yajing Lu, Yunxiao Yao, Lingjun Li

**Affiliations:** † Department of Chemistry, 5228 University of Wisconsin-Madison, Madison, Wisconsin 53706, United States; ‡ School of Pharmacy, 5228 University of Wisconsin-Madison, Madison, Wisconsin 53705, United States; § Lachman Institute for Pharmaceutical Development, School of Pharmacy, University of Wisconsin-Madison, Madison, Wisconsin 53705, United States; ∥ Wisconsin Center for NanoBioSystems, School of Pharmacy, University of Wisconsin-Madison, Madison, Wisconsin 53705, United States

## Abstract

High-throughput quantitative proteomics across diverse biological conditions, including varying perturbations and temporal changes, is critical for elucidating disease mechanisms and treatment responses. Advances in mass spectrometry and isobaric labeling technologies have enabled powerful multiplexed analyses; however, commonly used methods rely on low-mass reporter ions that are prone to ratio distortion due to peptide co-isolation and co-fragmentation in complex samples. Complementary ion-based quantification offers an alternative approach by leveraging peptide-specific fragment ions generated at the MS2 level, enabling more accurate and interference-resistant measurements. Here, we present a novel 7-plex dimethylated leucine complementary ion (DiLeuC) isobaric labeling strategy for sensitive and accurate multiplexed proteomics. Through systematic optimization of higher-energy collisional dissociation (HCD) and high-field asymmetric waveform ion mobility spectrometry (FAIMS), we significantly improved complementary ion generation efficiency and proteome coverage. The DiLeuC platform demonstrated high quantitative accuracy and precision across both defined-ratio samples and a human–yeast dual-proteome model, effectively quantifying low-abundance species in the presence of a high-abundance background. Overall, DiLeuC provides a robust and sensitive solution for multiplexed proteomics, enabling accurate complementary ion-based quantification at the MS2 level without requiring SPS-MS3 acquisition. Its efficient complementary ion generation and strong resistance to interference make it well suited for complex biological samples and high-throughput proteomic applications.

## Graphical abstract

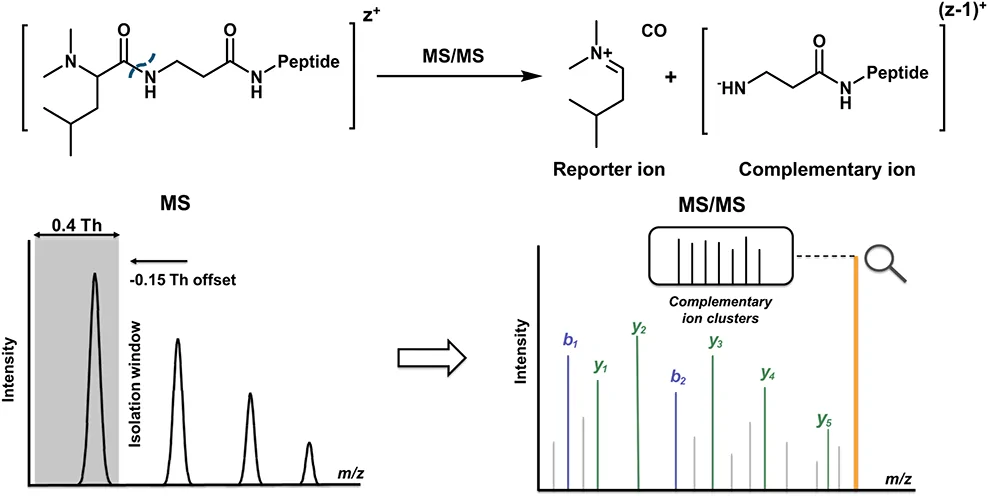

## 1 Introduction

Quantitative multiplexed proteomics has become an essential approach for analyzing proteome dynamics across multiple biological conditions, such as different time points or perturbations.^1–3^ This capability has been enabled by advances in mass spectrometry (MS) instrumentation and isobaric labeling strategies.^4,5^ In these workflows, proteins from different samples are enzymatically digested and labeled with distinct isobaric tags (e.g., TMT or iTRAQ), which encode quantitative information through the distribution of heavy isotopes between reporter and balancer groups.^6^ Because the total mass of each tag is identical, labeled peptides are indistinguishable at the MS1 level and can be analyzed simultaneously. Upon fragmentation during tandem MS (MS/MS), reporter ions are released in the low-mass region, enabling relative quantification by comparing their intensities. This multiplexing strategy offers significant advantages, including increased throughput, improved reproducibility, and reduced missing values due to co-analysis of samples within a single run.^7^ It is broadly applicable to diverse biological systems, as labeling is performed post-lysis, avoiding the need for metabolic incorporation of isotopes.^8^ Consequently, multiplexed proteomics has been widely adopted in studies ranging from cancer biology to fundamental cellular processes.^9,10^

Despite these advantages, a major challenge in isobaric labeling-based quantification is ratio distortion caused by the co-isolation and co-fragmentation of interfering peptide ions with similar mass-to-charge ratios.^11^ Because reporter ions derived from contaminants are indistinguishable from those of the target peptides, this interference compromises quantification accuracy and precision. This limitation is inherent to MS2-based quantification methods and was systematically demonstrated in early studies using mixed proteome systems.^12,13^

To address this issue, MS3-based strategies were developed, in which MS2 spectra are used for peptide identification and selected fragment ions are further fragmented to generate MS3 spectra for quantification.^14,15^ This additional gas-phase purification step significantly reduces interference and improves quantitative accuracy. However, conventional MS3 approaches suffer from reduced sensitivity, as only a small fraction of precursor ion signal is carried forward to the MS3 level.^16^ To mitigate this limitation, synchronous precursor selection (SPS)-MS3 was introduced, enabling simultaneous selection of multiple MS2 fragment ions for MS3 analysis, thereby substantially enhancing reporter ion signal intensity.^17,18^ While SPS-MS3 has become a widely adopted standard for improving quantification accuracy, it requires specialized instrumentation and still incurs a trade-off in sensitivity and duty cycle.^19–21^ As a result, developing strategies that maintain high quantitative accuracy while improving sensitivity and accessibility remains an important focus in multiplexed proteomics.

An alternative strategy to mitigate interference in multiplexed proteomics is based on complementary ions.^22,23^ Unlike SPS-MS3, complementary ion-based quantification operates at the MS2 level and does not require higher-order tandem MS acquisition. Therefore, it avoids the requirement for mass spectrometers capable of SPS-MS3 acquisition, such as the Orbitrap Fusion Lumos or Orbitrap Eclipse. In the present study, FAIMS was employed to further enhance complementary ion generation efficiency; however, complementary ions can also be generated and quantified without FAIMS. The principle of this strategy is that, during MS2 fragmentation of isobaric tag-labeled peptides, the cleavage that generates low-mass reporter ions simultaneously produces complementary ions that retain the peptide backbone along with the balancer region.^24^ These complementary ions carry channel-specific isotopic information analogous to reporter ions but differ in mass depending on the peptide sequence. As a result, they are precursor-specific and can distinguish co-isolated peptides, thereby substantially reducing ratio distortion compared to conventional MS2-based quantification.

This concept was first implemented in the TMTc method, in which the full isotopic envelope of precursor ions was isolated.^7,18^ However, this approach led to complex data deconvolution and limited quantitative accuracy. Subsequent improvements, including narrowing the isolation window and modeling its shape during data analysis, resulted in the enhanced TMTc+ approach, which significantly improved precision and accuracy. The strategy has since been extended to higher multiplexing capacity, including applications with TMTpro reagents.^25,26^

Despite its advantages, the efficiency of complementary ion formation depends strongly on tag design. Traditional isobaric tags such as iTRAQ^27^ and earlier *N*,*N*-dimethyl leucine (DiLeu)^28^ tags are not inherently suitable for this strategy because their fragmentation pathways do not retain sufficient structural features on the peptide fragment to generate distinguishable complementary ions.^1,29–31^ Existing TMT and TMTpro reagents also exhibit relatively low complementary ion formation efficiency, which limits the practical sensitivity of the approach. To address this limitation, our group previously developed a modified DiLeu tag, termed DiLeuC, by introducing a β-alanine linker that promotes the formation of informative peptide-specific complementary ions.^32^

In our previous proof-of-concept study, DiLeuC demonstrated the feasibility of accurate complementary ion–based quantification at the MS2 level.^32^ However, practical implementation of the complete 7-plex reagent set was limited by incomplete ^18^O incorporation in channels 7–6 and 7–7, which generated substantial ^18^O-deficient isotopologues and restricted the initial application to four channels. In the present study, we address this limitation by redesigning and optimizing the ^18^O-exchange and NHS activation procedures to achieve high isotopic purity across all seven DiLeuC channels. We further establish and systematically optimize the complete 7-plex DiLeuC workflow, including HCD fragmentation and FAIMS conditions, and evaluate its quantitative accuracy and precision using defined-ratio samples and a complex human–yeast two-proteome model.

Importantly, complementary ion-based quantification maintains high sensitivity because it operates entirely at the MS2 level without requiring additional fragmentation steps. With optimized sample preparation workflows and instrument parameters, this approach provides a streamlined and accurate alternative to MS3-based methods. By leveraging efficient fragmentation and flexible tag design, DiLeuC has further demonstrated strong potential for highly sensitive applications.

## 2 Materials and Methods

### 2.1 Chemicals and Materials

Acetic acid (AA), acetonitrile (ACN), acetone, dimethyl sulfoxide (DMSO), formic acid (FA), methanol (MeOH), TMTpro reagent, and water were obtained from Fisher Scientific (Pittsburgh, PA). Beta-alanine and its isotopically labeled derivatives (^15^N-beta-alanine, 3-^13^C–^15^N-beta-alanine, 1,2-^13^C_2_-^15^N-beta-alanine, and ^13^C_3_-^15^N-beta-alanine), l-leucine and its labeled forms (1-^13^C-leucine and 1-^13^C, ^15^N-leucine), formaldehyde and ^13^C-formaldehyde, sodium cyanoborohydride, ^18^O water, 1-ethyl-3-(3-(dimethylamino)­propyl)­carbodiimide (EDCI), hydroxybenzotriazole (HOBt), and *N*-hydroxysuccinimide (NHS) were purchased from Sigma-Aldrich (St. Louis, MO). Trypsin, LysC, and yeast protein digests were obtained from Promega Corporation (Madison, WI). Ethylene-bridged hybrid C18 stationary phase material was purchased from PolyLC Inc. (Columbia, MD). Fused silica capillary tubing (75 μm i.d., 375 μm o.d.) was obtained from Polymicro Technologies (Phoenix, AZ). All reagents were used as received without further purification.

### 2.2 Proteomics Sample Preparation

K562 protein extracts were reconstituted in 8 M urea with 50 mM TEAB buffer (pH 8.0). Samples were reduced with 5 mM DTT at 37 °C for 2 h, followed by alkylation with 15 mM iodoacetamide (IAA) at room temperature in the dark for 30 min. Excess IAA was quenched by adding 5 mM DTT and incubating for an additional 10 min. The urea concentration was then diluted to 1.6 M using 50 mM TEAB buffer. Proteins were digested with trypsin at an initial protein-to-enzyme ratio of 100:1 and incubated at 37 °C. After 12 h, an additional aliquot of trypsin was added to achieve a final ratio of 50:1, followed by further incubation at 37 °C for 4 h. Digestion was terminated by adding trifluoroacetic acid (TFA) to a final concentration of 1%. The resulting peptides were desalted using Sep-Pak C18 cartridges and subsequently dried under vacuum.

### 2.3 DiLeuC Labeling

The detailed synthetic workflow for the 7-plex DiLeuC reagents is provided in the Supporting Information. Peptide concentrations were determined using a BCA assay, and equal amounts of peptides were aliquoted for each DiLeuC labeling channel. 7-plex DiLeuC tags were dissolved in anhydrous acetonitrile (ACN) at a concentration of 4 μg/μL. Digested peptides were reconstituted in 100 mM triethylammonium bicarbonate (TEAB) buffer and labeled at a peptide-to-tag ratio of 1:10. The labeling reaction was carried out at room temperature for 1 h. The reaction was then quenched by adding 5% hydroxylamine and incubating for an additional 15 min at room temperature. Samples labeled with different channels were subsequently combined and dried under vacuum using a SpeedVac. The pooled sample was reconstituted in 150 μL of 5% trifluoroacetic acid (TFA), desalted using Sep-Pak C18 cartridges, and dried again in vacuo. Finally, peptides were reconstituted in water containing 0.1% formic acid (FA) for LC–MS/MS analysis.

### 2.4 Offline HpH Fractionation

High-pH reversed-phase (HpH) fractionation was performed on a Waters Alliance e2695 HPLC using a 150 × 2.1 mm, 5 μm, 100 Å C18 column (Phenomenex) at a flow rate of 0.2 mL/min. Mobile phase A consisted of 10 mM ammonium formate in water (pH 10), and mobile phase B consisted of 10 mM ammonium formate in 90% acetonitrile (ACN) (pH 10). Peptide separation was carried out using the following gradient: 1% B (0–5 min), 1–40% B (5–50 min), 40–60% B (50–54 min), 60–70% B (54–58 min), and 70–100% B (58–59 min). Fractions were collected at 4 min intervals, yielding a total of 14 fractions. Nonadjacent fractions were subsequently concatenated into four pooled samples for LC–MS/MS analysis.

### 2.5 LC-MS/MS Analysis

Nano LC–MS/MS analysis was performed using a Vanquish Neo UHPLC coupled to an Orbitrap Exploris 480 and equipped with a FAIMS Pro DUO interface (Thermo Fisher Scientific, Bremen, Germany). MS1 spectra were acquired in the Orbitrap over an m/z range of 400–2000 at a resolution of 120,000, with an automatic gain control (AGC) target of 4 × 10^5^ and a maximum injection time of 50 ms. The RF lens was set to 60%, and dynamic exclusion was applied for 30 s across all charge states of a given precursor. Tandem MS (MS/MS) data were collected in data-dependent acquisition (DDA) mode with a first mass of m/z 100, a resolution of 60,000, and a normalized collision energy of 30. A narrow asymmetric isolation window of 0.4 Th with a −0.15 Th offset was applied to selectively isolate the monoisotopic precursor peak, thereby minimizing complementary ion cluster overlap from co-isolated isotopic peaks and simplifying downstream deconvolution (Figure 1D). Chromatographic separation was carried out using mobile phase A (0.1% formic acid in water) and mobile phase B (0.1% formic acid in acetonitrile). Peptides were loaded onto a 75 μm × 15 cm in-house packed column containing 1.7 μm, 130 Å BEH C18 material (Waters, part no. 186004661). Emitter tips were fabricated from 75 μm i.d. fused silica capillaries using a P-2000 laser puller (Sutter Instrument Co., Novato, CA). The LC gradient was as follows: 3–10% B (18–31 min), 10–24% B (31–95 min), and 24–35% B (95–127 min), with a flow rate of 300 nL/min.

**Figure 1:**
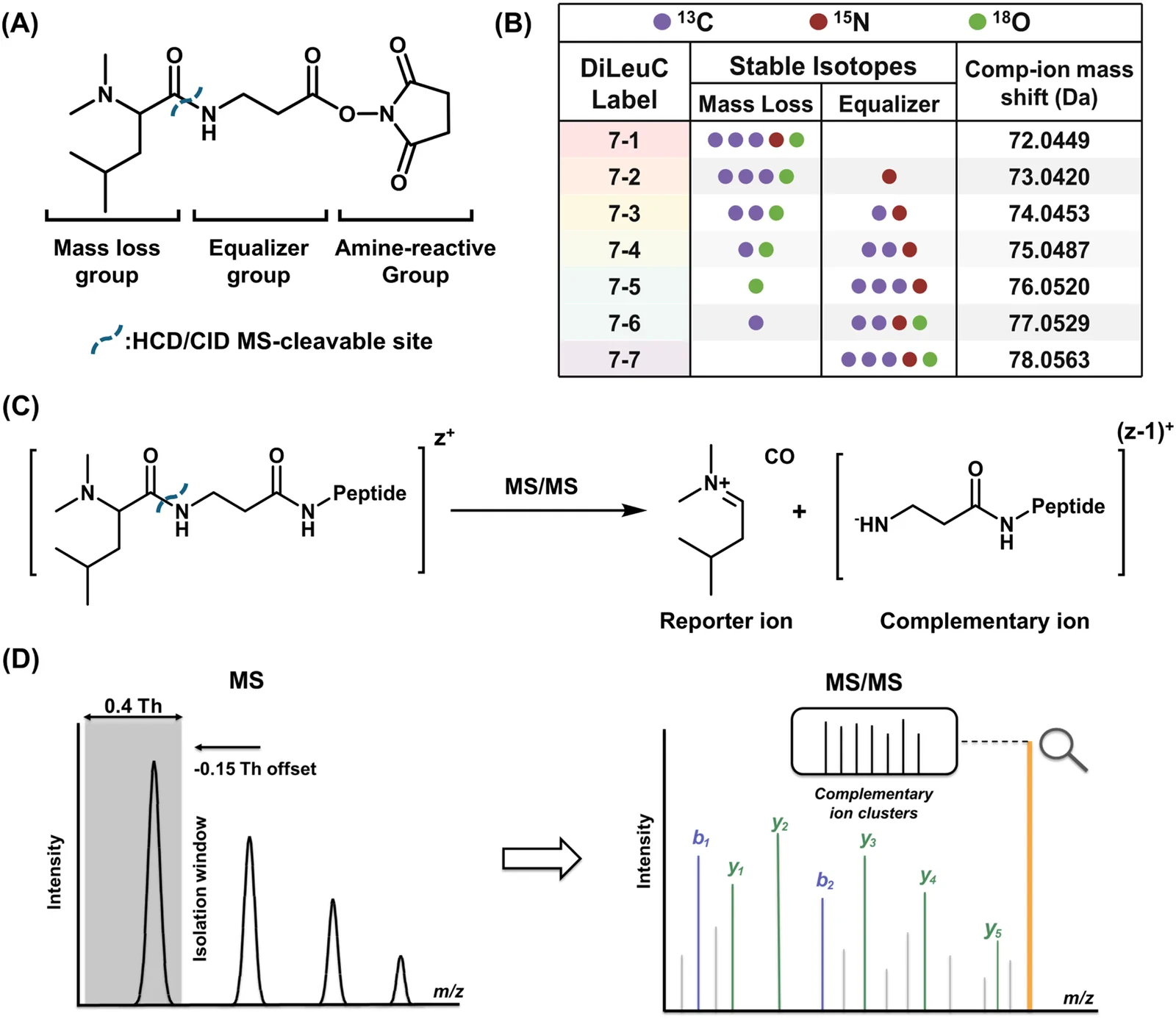
(A) Chemical structure of the DiLeuC tag, comprising a mass-loss group, an equalizer group, and an amine-reactive NHS ester group. The HCD/CID-cleavable site is indicated by the dashed blue line. (B) Isotopic distributions and complementary-ion mass shifts of the 7-plex DiLeuC tag. (C) Upon MS/MS fragmentation, DiLeuC-labeled peptides produce a low-mass reporter ion and a peptide-containing complementary ion that retains channel-specific isotopic information. (D) A narrow, asymmetric precursor isolation window selectively transmits the monoisotopic peak while minimizing isotopic interference, enabling accurate complementary ion-based quantification from the MS/MS spectrum.

### 2.6 Data Analysis

Data analysis was performed using MSFragger (version 4.3) within FragPipe (version 23.1).^33,34^ For K562 and PANC-1 datasets, the reviewed *Homo sapiens* proteome database (UniProt UP000005640; downloaded October 7, 2025; 40,906 entries including 20,453 decoys) was used. Yeast datasets were searched against the *Saccharomyces cerevisiae* proteome database (UniProt UP000002311; downloaded April 28, 2025; 12,366 entries including 6,183 decoys). Precursor and fragment mass tolerances were set to ±20 ppm, and a minimum peptide length of seven amino acids was required. Carbamidomethylation of cysteine (+57.02146 Da) and either DiLeuC zero or DiLeuC labeling on lysine residues and peptide N-termini were specified as static modifications, while methionine oxidation (+15.99492 Da) and protein N-terminal acetylation were set as dynamic modifications. Results were filtered to a 1% false discovery rate (FDR) at both the peptide and protein levels.

FragPipe search outputs were further processed for complementary ion analysis using an in-house Python script provided as Supporting Information. The script calculates theoretical complementary ion *m/z* values based on precursor *m/z* and charge state, matches complementary ions in the corresponding MS2 spectra using a 20 ppm mass tolerance, and extracts their intensities for downstream quantification. Raw mass spectrometry files (.raw) were converted to.mzML format using MSConvertGUI.^35^ Peptide-spectrum matches (PSMs) and protein assignments were obtained from the exported PSM.tsv file. Theoretical *m/z* values for complementary ions were calculated based on matched peptide masses, and a 20 ppm tolerance was applied for matching. Using scan numbers, PSMs were linked to corresponding complementary ion intensities, which were then used for peptide- and protein-level quantification. MS spectra were visualized and inspected using Xcalibur (version 4.0), and graphical and statistical analyses were performed with GraphPad Prism (version 8.0.2).

## 3 Results and Discussion

### 3.1 Resolving the ^18^O-Exchange Problem Enables the Complete 7-Plex DiLeuC Reagent Set

Complementary ion-based quantification provides an attractive MS2-level strategy for multiplexed proteomics because it reduces coisolation-induced ratio compression without requiring the additional gas-phase purification steps used in MS3-based workflows. In this strategy, activation of an isobaric tag-labeled peptide generates a precursor-specific peptide–equalizer complementary ion that retains the channel-encoded isotopic information within a high-mass, analyte-dependent *m/z* window (Figure 1A–C). This high-mass complementary ion is precursor-specific and preserves quantitative information even when unrelated peptides are co-isolated and co-fragmented (Figure 1D).

Most complementary-ion reagent designs have relied primarily on^13^C and ^15^N isotope encoding. In principle, ^18^O is particularly attractive for expanding multiplexing capacity because each substitution contributes an approximately 2 Da mass shift, enabling larger channel spacing with fewer heavy atoms. ^18^O was incorporated into the design of our DiLeuC tag; however, practical implementation of the full reagent set was limited by incomplete ^18^O incorporation in channels 7–6 and 7–7, in which ^18^O is encoded within the β-alanine equalizer region (Figure 1 and Figure S1). High-resolution MS analysis showed that the ^18^O-deficient isotopologue accounted for more than 30% of the reagent signal in these channels in previous work.^32^ This incomplete ^18^O incorporation is likely caused by oxygen exchange during NHS ester activation. In addition, the original synthetic route introduced ^18^O exchange before benzyl protection and deprotection, leaving the labeled carboxylate susceptible to reverse exchange during subsequent synthetic manipulations. Because isotopic defects in complementary-ion reagents directly redistribute signals among channel-specific complementary-ion clusters and generate channel cross-talk, this level of impurity was incompatible with accurate quantification. Consequently, the previous proof-of-concept study was restricted to four channels, leaving the full 7-plex capability of DiLeuC unrealized.^32^

To overcome this limitation, we redesigned the synthesis at two key stages: the ^18^O incorporation and the subsequent active-ester formation step. First, ^18^O exchange was moved to a later stage of the synthesis, and the ^18^O exchange workflow was optimized. Briefly, after *N*,*N*-dimethyl leucine−β-alanine intermediate was synthesized, the material was subjected to an iterative evaporative ^18^O-exchange workflow (Figure S2). In each cycle, the intermediate was exchanged with excess H_2_^18^O and then dried *in vacuo*. Fresh H_2_^18^O was added for the next cycle, and the exchange–evaporate process was repeated three times. This procedure continuously replaces the exchange medium and removes generated H_2_^16^O, thereby driving the oxygen-exchange equilibrium toward the ^18^O-enriched product more effectively than a single static incubation in H_2_^18^O. A second modification was introduced during NHS activation. Because the ^18^O labels reside on the carboxylate-derived oxygen atoms, they are susceptible to reverse exchange during active-ester formation. To suppress this scrambling, NHS esterification was performed in the presence of 3% (v/v) H_2_^18^O in ACN. Maintaining an ^18^O-rich environment during this step minimizes exchange with residual H_2_^16^O.

High-resolution MS analysis of the purified reagents confirmed that the redesigned synthesis substantially improved ^18^O incorporation in channels 7–6 and 7–7, increasing the isotopic purity from below 70% in the original workflow to greater than 95% in the optimized workflow (Figure S3). Importantly, this improved ^18^O enrichment was also preserved after labeling and complementary-ion generation. Because complementary ions cannot be directly observed in the MS/MS spectrum of the tag, ^13^C_6_-arginine was labeled with the seven DiLeuC channels and then mixed at near equal ratios as a model system to evaluate full 7-plex complementary-ion readout. Upon MS/MS fragmentation, the labeled ^13^C_6_-arginine generated seven complementary ions approximately 1 Da apart (Figure S4). The corresponding equalizer-dependent mass increment ranged from 72 to 78 Da after subtracting the arginine mass. The nearly uniform intensities across all seven complementary-ion channels confirmed that the optimized synthesis minimized ^18^O-related isotopic defects, thereby enabling accurate 7-plex DiLeuC-based quantification.

### 3.2 Optimization of HCD Collision Energy and FAIMS Compensation Voltage for Improved Proteome Coverage and Complementary Ion Generation

We optimized higher-energy collisional dissociation (HCD) fragmentation energy to maximize proteome coverage. Protein and peptide identification and quantification metrics were evaluated across different conditions, as illustrated in Figure S5. Tryptic digests of K562 cells were labeled with DiLeuC zero, and proteome coverage was assessed at normalized collision energies (NCE) of 26%, 28%, 30%, 32%, and 34%. As shown in Figure 2A, the numbers of both identified and quantified proteins peaked at NCE 30%, indicating the highest proteome coverage under this condition. A similar trend was observed at the peptide level, where NCE 30% resulted in 1,451 peptide identifications, of which 1,367 were quantifiable via complementary ions (Figure 2B). This condition yielded the greatest number of both protein and peptide quantifications among all tested settings. Together, these results demonstrate that NCE 30% provides optimal performance for complementary ion–based quantification, and this setting was therefore used for all subsequent experiments. Representative MS/MS spectra acquired at NCE 26%, 28%, 30%, 32%, and 34% are shown in Figure S6. Complementary ions were consistently observed across the evaluated HCD energy range, with relatively similar complementary ion patterns under the different NCE conditions. These spectra further demonstrate the robust generation of DiLeuC complementary ions across a broad range of collision energies.

**Figure 2:**
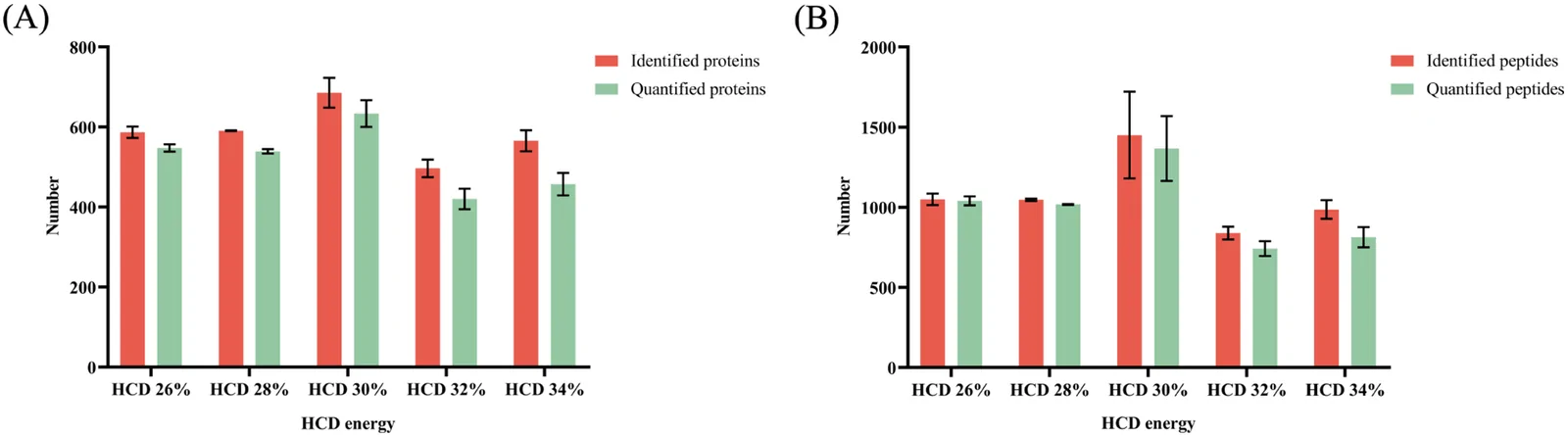
(A) Protein-level results under different NCE conditions. The bar graph shows the total number of identified proteins (red) and quantified proteins using complementary ions (green). (B) Peptide-level results under different NCE conditions. The bar graph shows the total number of identified peptides (red) and quantified peptides using complementary ions (green). Error bars represent the standard deviation across technical replicates.

We next evaluated the use of high-field asymmetric waveform ion mobility spectrometry (FAIMS) to enhance complementary ion generation.^36–38^ To identify optimal conditions, the FAIMS compensation voltage (CV) was systematically optimized by monitoring the complementary ion generation efficiency. Tryptic digests of K562 cells labeled with DiLeuC zero were analyzed at CV settings of −35, −45, −55, −65, −75, as well as in combined CV modes (−35/–55, −45/–65, −55/–75, and −30/–55/–70). As shown in Figure 3A, complementary ion generation efficiency—defined as the percentage of peptide-spectrum matches (PSMs) containing quantifiable complementary ions relative to the total identified PSMs—reached a maximum of 85% at CV −45/–65. This condition yielded the highest proportion of complementary-ion-containing PSMs. In addition, comparison of experiments performed with and without FAIMS (Figure 3B) demonstrated that FAIMS substantially improves complementary ion generation efficiency. Together, these results indicate that the CV −45/–65 setting provides optimal performance, and this condition was therefore applied in all subsequent experiments. The absolute numbers of identified PSMs and PSMs containing quantifiable complementary ions under each FAIMS condition are provided in Table S1. These values complement the percentages shown in Figure 3A and provide a more comprehensive assessment of the effects of FAIMS on peptide identification and complementary ion generation. To further evaluate whether FAIMS compromised signal intensity through reduced ion transmission, signal intensity distributions were compared between the no-FAIMS and optimized FAIMS conditions (Figure S7). The median log2 signal intensity was approximately 14.3 without FAIMS and 16.5 with FAIMS, with substantial overlap between the two distributions. These results indicate that the optimized FAIMS conditions did not result in an apparent loss of signal intensity in this dataset while improving complementary ion generation efficiency.

**Figure 3:**
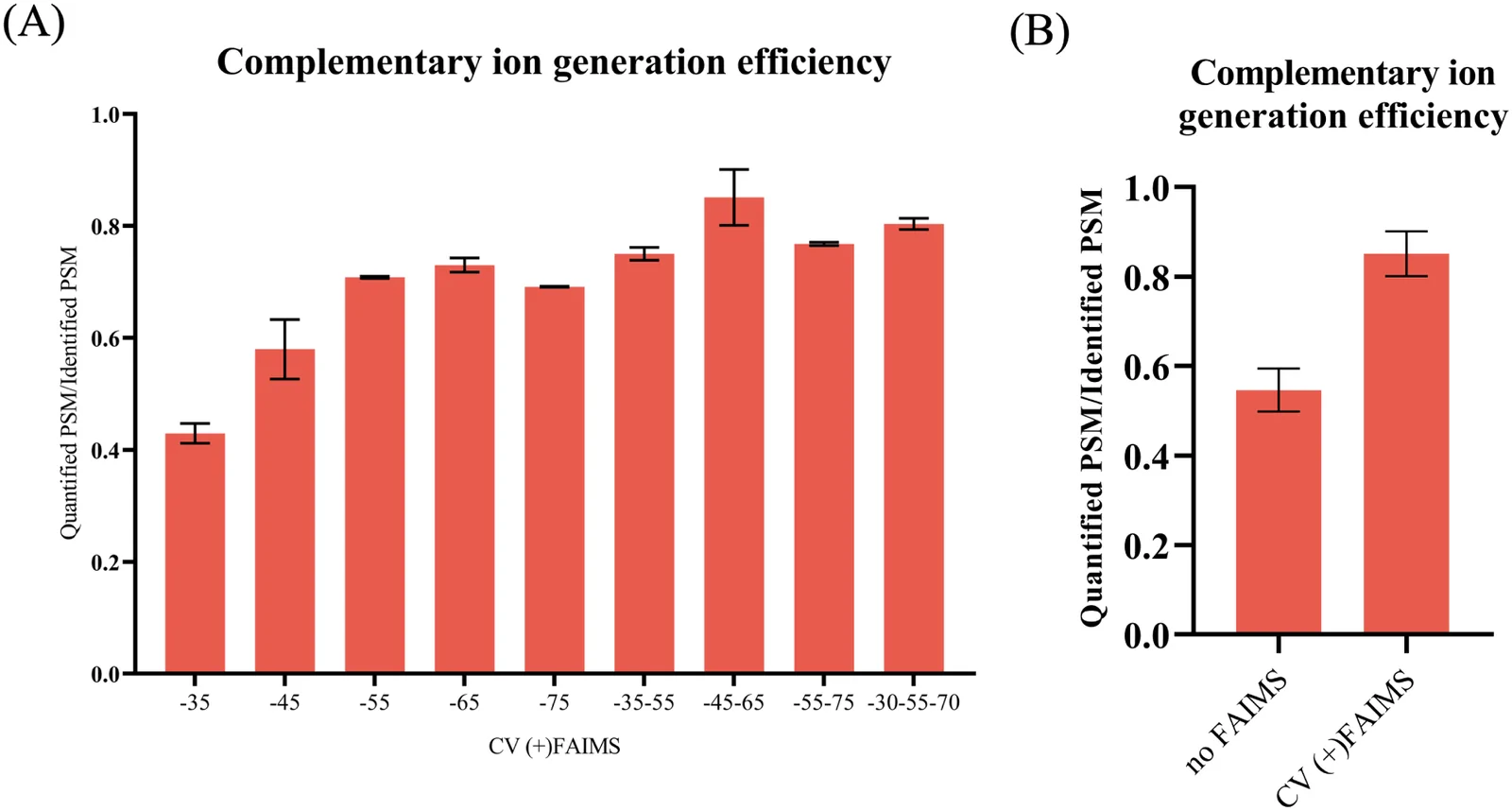
Evaluation and optimization of FAIMS CV settings for complementary ion generation efficiency. (A) Ratio of quantified PSMs to identified PSMs across different CV conditions. (B) Comparison of complementary ion generation efficiency with and without FAIMS.

High-pH (HpH) fractionation is a widely used offline peptide separation strategy in proteomics that reduces sample complexity prior to LC–MS/MS analysis, thereby enhancing proteome coverage.^39,40^ This approach is well suited for DiLeuC workflows, as it decreases sample complexity while preserving high sensitivity. Following HpH fractionation, a total of 18,296 peptides corresponding to 3,609 proteins were identified from 7-plex DiLeuC-labeled tryptic digests of K562 cells, of which 17,852 peptides were successfully quantified (Figure S8).

The performance of the newly developed DiLeuC reagents was evaluated against established TMTproC reagents. Comparisons were performed under identical experimental conditions using tryptic digests of K562 cells. As shown in Figure S9A, peptides were labeled with either DiLeuC zero or TMTpro zero reagents, followed by cleanup and LC–MS/MS analysis. The results demonstrate that DiLeuC labeling achieves higher complementary ion generation efficiency compared to TMTproC labeling (Figure S9B).

### 3.3 Quantitative Analysis of 7-Plex DiLeuC-Labeled PANC-1 Samples

Building on the optimized HCD fragmentation energy and FAIMS CV settings, we applied the DiLeuC workflow to evaluate its quantitative accuracy and precision. Tryptic digests of PANC-1 cells were labeled with 7-plex DiLeuC reagents and mixed at defined ratios of 1:1:1:1:1:1:1 and 1:2:5:10:5:2:1 (channels 7–1 to 7–7). The samples were analyzed by LC–MS/MS, and the measured ratios were compared with the theoretical values to assess quantification performance. As shown in Figure 4, the measured abundances closely matched the expected ratios, demonstrating the high quantitative accuracy of the 7-plex DiLeuC system in complex proteomic samples. The distribution of quantified signals reflected the designed mixing ratios, with median coefficients of variation (CVs) of 13% for the 1:1:1:1:1:1:1 sample and 20% for the 1:2:5:10:5:2:1 sample. These results indicate robust precision and reliable quantitative performance. A total of 595 peptides and 1022 peptides were quantified and included in the analysis of the 1:1:1:1:1:1:1 and 1:2:5:10:5:2:1 PANC-1 samples, respectively. To evaluate the influence of charge state on DiLeuC quantification using a fixed isolation window, the equal-ratio PANC-1 dataset was further analyzed separately for 2+ and 3+ ions (Figure S10). Both charge states produced median ratios close to the expected 1:1 ratio across all seven channels. The 2+ precursors generally exhibited tighter quantitative distributions than the 3+ precursors, although both charge states maintained good overall quantitative accuracy. These results demonstrate that the fixed isolation-window strategy supports reliable complementary ion quantification across the predominant 2+ and 3+ precursor populations.

**Figure 4:**
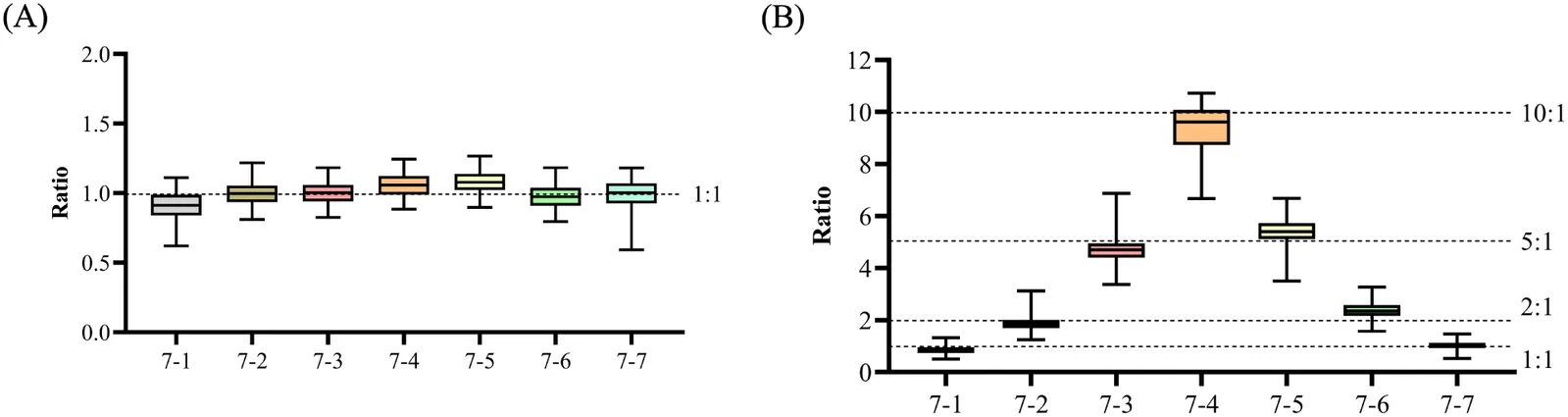
Quantitative accuracy of 7-plex DiLeuC-labeled samples. Tryptic peptides derived from PANC-1 cells were labeled with 7-plex DiLeuC reagents and combined at ratios of 1:1:1:1:1:1:1 or 1:2:5:10:5:2:1. The mixtures were analyzed by LC–MS/MS at a resolving power of 60,000. Peptide-level quantitative ratios were determined for (A) the equimolar (1:1:1:1:1:1:1) sample and (B) the gradient (1:2:5:10:5:2:1) sample. Box plots display the median (center line), interquartile range (25th–75th percentiles, box), and the 5th–95th percentile range (whiskers).

### 3.4 DiLeuC-Based Analysis of a Two-Proteome Model

After validating the quantitative performance of 7-plex DiLeuC in defined-ratio PANC-1 peptide mixtures, we next evaluated whether the method could maintain quantitative accuracy in a more challenging two-proteome model. A human–yeast two-proteome model was designed using K562 (human) and *Saccharomyces cerevisiae* (yeast) lysates. K562 peptides were labeled with 7-plex DiLeuC tags at a 1:1:1:1:1:1:1 ratio, whereas yeast peptides were labeled at a 1:2:5:10:5:2:1 ratio (channels 7–1 to 7–7). The labeled samples were then combined at a yeast-to-K562 ratio of 1:5 to mimic the quantification of low-abundance yeast peptides in a high-abundance human peptide background. The mixture was analyzed by LC–MS/MS on an Orbitrap Exploris 480 (Figure 5A). This model provides a stringent test for complementary ion-based quantification because coisolated human peptides can contribute substantial background signals during MS/MS acquisition. For conventional low-mass reporter ion quantification, such interference would be expected to compress the measured yeast ratios toward the constant human background.

**Figure 5:**
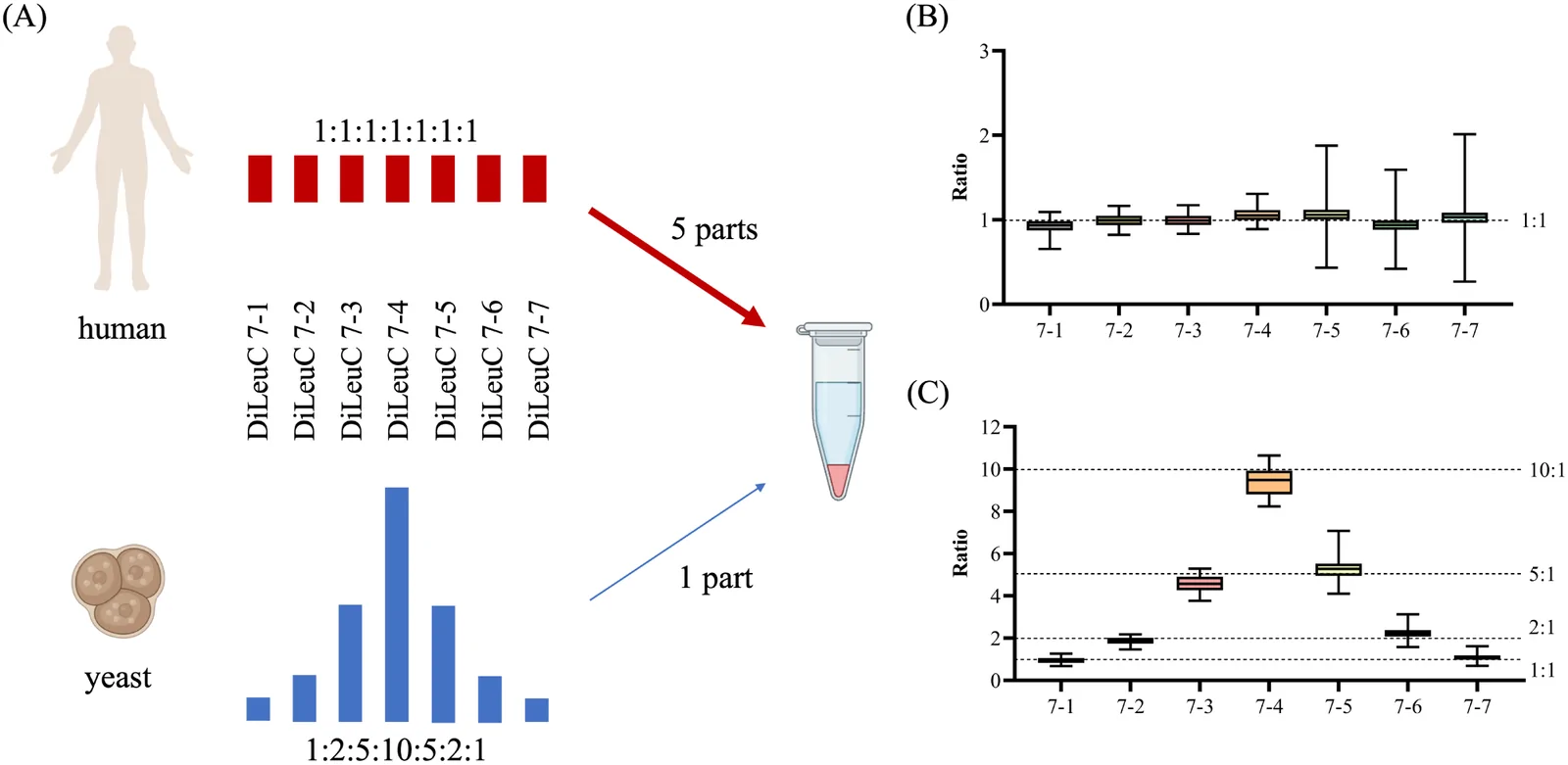
Quantitative accuracy and precision of DiLeuC in a human–yeast dual-proteome model. (A) Schematic illustration of the experimental design. K562 peptides were labeled with DiLeuC tags at a 1:1:1:1:1:1:1 ratio (channels 7–1 to 7–7), whereas yeast peptides were labeled at a 1:2:5:10:5:2:1 ratio. The labeled samples were then combined at a yeast-to-K562 ratio of 1:5. (B) Box plots showing quantified ratios for human proteins. (C) Box plots showing quantified ratios for yeast proteins. Dashed lines indicate the theoretical ratios.

For K562 proteins, the median quantified ratios were 0.93:0.99:0.99:1.05:1.06:0.94:1.03, whereas yeast proteins exhibited median ratios of 0.93:1.89:4.57:9.48:5.27:2.22:1.09. In both cases, the measured values closely matched the expected theoretical ratios (Figure 5B and C). A total of 1796 human peptides and 710 yeast peptides were quantified and included in the quantitative distributions shown in Figure 5B and 5C, respectively. The accurate recovery of both the unchanged K562 background and the differentially mixed yeast proteome demonstrates that the 7-plex DiLeuC workflow preserves quantitative accuracy in complex two-proteome samples.

## 4 Conclusions

In this study, we developed a novel 7-plex DiLeuC isobaric labeling strategy that integrates the advantages of multiplexed proteomics with the interference resistance of complementary ion-based quantification. The redesigned synthetic workflow overcame the ^18^O-exchange bottleneck, achieving high isotopic purity across the complete 7-plex DiLeuC reagent set and ensuring accurate quantification. The DiLeuC design promotes efficient generation of informative complementary ions, addressing the low complementary-ion yield and high reagent cost associated with existing isobaric tags while improving MS2-level quantification accuracy. Systematic optimization of HCD collision energy and FAIMS CV significantly enhanced both proteome coverage and complementary ion generation efficiency. The optimized workflow demonstrated robust performance, achieving high identification rates. Compared with existing complementary ion approaches, DiLeuC exhibited improved complementary ion generation efficiency, highlighting its effectiveness for accurate multiplexed quantification. The 7-plex DiLeuC platform showed excellent quantitative accuracy and precision across both simple and complex samples, as validated using PANC-1 peptide mixtures and a human–yeast dual-proteome model. Accurate quantification was maintained even in the presence of a high-abundance background, demonstrating the robustness of the method for challenging biological samples. Overall, the DiLeuC strategy provides a sensitive and accurate solution for multiplexed proteomics by enabling complementary ion–based quantification at the MS2 level without requiring SPS-MS3 acquisition. Although FAIMS was employed in the optimized workflow to enhance complementary ion generation efficiency, DiLeuC complementary ions can also be generated and quantified without FAIMS. The efficient complementary ion generation, streamlined data analysis, and strong quantitative performance of DiLeuC make it a promising tool for a wide range of proteomic applications.

## Supplementary Material





## Notes

Chemical synthetic route of DiLeuC isobaric tag (Scheme S1); Isotopic distribution of the 7-plex DiLeuC isobaric tag (Figure S1); iterative 18O-exchange workflow (Figure S2); high-resolution MS characterization of optimized DiLeuC 7–6 and 7–7 reagents (Figure S3); MS/MS fragmentation scheme and MS/MS spectrum (Figure S4); workflow of the DiLeuC strategy (Figure S5); representative MS/MS spectra illustrating DiLeuC complementary ion generation across different HCD collision energies (Figure S6); comparison of signal intensity with and without FAIMS (Figure S7); number of identified and quantified proteins and peptides in K562 cells (Figure S8); comparison of complementary ion generation efficiency between DiLeuC zero and TMTpro zero methods (Figure S9); charge-state-specific quantitative performance of the 7-plex DiLeuC (Figure S10); Identification and complementary ion quantification performance under different FAIMS conditions. (Table S1); comparison of quantitative accuracy and precision reported for complementary-ion- and reporter-ion-based isobaric quantification strategies (Table S2) (PDF)
DiLeuC complementary ion analysis (PDF)
