# Supplementary material for: Accurate and Sensitive Multiplexed Proteomics Using the 7‑Plex DiLeuC Complementary Ion Strategy
Source: J Am Soc Mass Spectrom. 2026 Oct 6;37(11):2759–67. doi: 10.1021/jasms.6c00234 (PMC13741911; doi:10.1021/jasms.6c00234)
Supplement: Supplementary file 1 [file js6c00234_si_001.pdf]

Supporting Information

Accurate and Sensitive Multiplexed Proteomics Using 7-plex DiLeuC Complementary Ion Strategy

Jingwei Zhang[1,#], Zicong Wang[2,#], Yajing Lu[1], Yunxiao Yao[1], Lingjun Li[1,2,3,4*]

[1] Department of Chemistry, University of Wisconsin-Madison, Madison, WI, 53706, USA

[2] School of Pharmacy, University of Wisconsin-Madison, Madison, WI, 53705, USA

[3] Lachman Institute for Pharmaceutical Development, School of Pharmacy, University of Wisconsin-Madison, Madison, WI, 53705, USA

[4] Wisconsin Center for NanoBioSystems, School of Pharmacy, University of Wisconsin-Madison, Madison, WI 53705, USA.

[#3] These authors contributed equally: Jingwei Zhang and Zicong Wang

*Corresponding author: Lingjun Li

Email: lingjun.li@wisc.edu

Mailing address: 777 Highland Ave, Madison, WI 53705, United States

Tel: +1 608-265-8491, Fax: +1 608-262-5345

Table of Contents

Scheme S1. Chemical synthetic route of DiLeuC isobaric tag

Figure S1. Isotopic distribution of 7-plex DiLeuC isobaric tag

Figure S2. Iterative ^{18}O-exchange workflow

Figure S3. High-resolution MS characterization of optimized DiLeuC 7-6 and 7-7 reagents

Figure S4. MS/MS fragmentation scheme and MS/MS spectrum

Figure S5. Workflow of the DiLeuC Strategy

Figure S6. Representative MS/MS spectra illustrating DiLeuC complementary ion generation across different HCD collision energies.

Figure S7. Comparison of signal intensities with and without FAIMS.

Figure S8. Number of identified and quantified proteins and peptides in K562 cells

Figure S9. Comparison of complementary ion generation efficiency between DiLeuC zero and TMTpro zero methods

Figure S10. Charge-state-specific quantitative performance of 7-plex DiLeuC.

Table S1. Identification and complementary ion quantification performance under different FAIMS conditions.

Table S2. Comparison of quantitative accuracy and precision reported for complementary ion– and reporter ion–based isobaric quantification strategies.

Chemical Synthesis of DiLeuC Isobaric Tags

L-Leucine (100mg, 0.76mmol) and sodium cyanoborohydride (120mg, 1.91mmol) were mixed in 20ml MeOH under ice bath, then 275uL formaldehyde (37% w/w. 3.8mmol) was added. The mixture was stirred at rt for 12 h. After the reaction was complete, the solvent was removed and then purified by column chromatography on silica gel using DCM/Methanol (5% to 40% methanol) to yield N, N-dimethyl-leucine (110mg, 91%) as white solid.

β-Alanine (100 mg, 1.12 mmol) and p-toluenesulfonic acid monohydrate (235 mg, 1.23 mmol) were suspended in 10 mL toluene, then benzyl alcohol (128 μL, 1.23 mmol) was added. The mixture was refluxed at 110 °C for 12 h. After the reaction was complete, the mixture was cooled to room temperature, diluted with EtOAc (30 mL), washed with saturated aqueous $NaHCO_3$ and brine, dried over anhydrous Na_2SO_4, filtered, and concentrated to yield β-alanine benzyl ester p-toluenesulfonate (374 mg, 95%) as a white solid.

N,N-Dimethyl-leucine (100 mg, 0.63 mmol, 1.0 eq), EDCI (144 mg, 0.75 mmol, 1.2 eq), HOBt (102 mg, 0.75 mmol, 1.2 eq), β-alanine benzyl ester p-toluenesulfonate (242 mg, 0.69 mmol, 1.1 eq), and NMM (276 μL) were mixed in 20 mL DCM. The mixture was stirred at rt for 16 h. After the reaction was complete, the mixture was concentrated. The residue was purified by column chromatography on silica gel using DCM/Methanol (0% to 10% methanol) to yield N,N-Dimethyl-leucine- β-Alanine benzyl ester (171 mg, 85%) as oil-like product.

N,*N*-Dimethyl-leucine-β-Alanine benzyl ester (171 mg, 0.53 mmol) was dissolved in 10 mL MeOH, then 10% Pd/C (17 mg) was added. The mixture was stirred under 100 bar H_2 at 50C for 4 h. After the reaction was complete, the mixture was filtered through Celite, washed with MeOH, and the filtrate was concentrated under reduced pressure. The residue was purified by column

chromatography on silica gel using DCM/Methanol (5% to 40% methanol) to yield N,N-Dimethyl-leucine-β-Alanine (117 mg, 95%) as white solid.

N,*N*-Dimethyl-leucine-β-Alanine (117 mg, 0.51 mmol, 1.0 eq), N-hydroxysuccinimide (70 mg, 0.61 mmol, 1.2 eq), and EDCI (117 mg, 0.61 mmol, 1.2 eq) were mixed in 10 mL ACN. For ^{18}O exchanged *N*,*N*-Dimethyl-leucine-β-Alanine, 300 uL $H_2{}^{18}O$ was added. The mixture was stirred at rt for 4 hours. After the reaction was completed, the solvent was removed under reduced pressure and the residue was purified by column chromatography on silica gel using DCM/(ACN: Acetone = 1:1) to yield *N*,*N*-Dimethyl-leucine-β-Alanine NHS ester (105 mg, 63%) as oil-like product. The product was completely dried and stored in -20℃ until use.

^{18}O Exchange Workflow

100mg N,N-Dimethyl-leucine-β-Alanine or β-Alanine was transferred into a 2-ml Eppendorf tube, then 1.5ml $H_2{}^{18}O$ was added. For β-Alanine, the $H_2{}^{18}O$ was acidified using HCl (g) as described in Frost et al.[1] to enhance the exchange rate. The tube was sealed and incubated at 60 °C for 4 hours. The solution was then dried in vacuo, and another 1.5 mL $H_2{}^{18}O$ was added. This cycle was repeated three times. After the final cycle, the product was completely dried and immediately used in the subsequent coupling step.

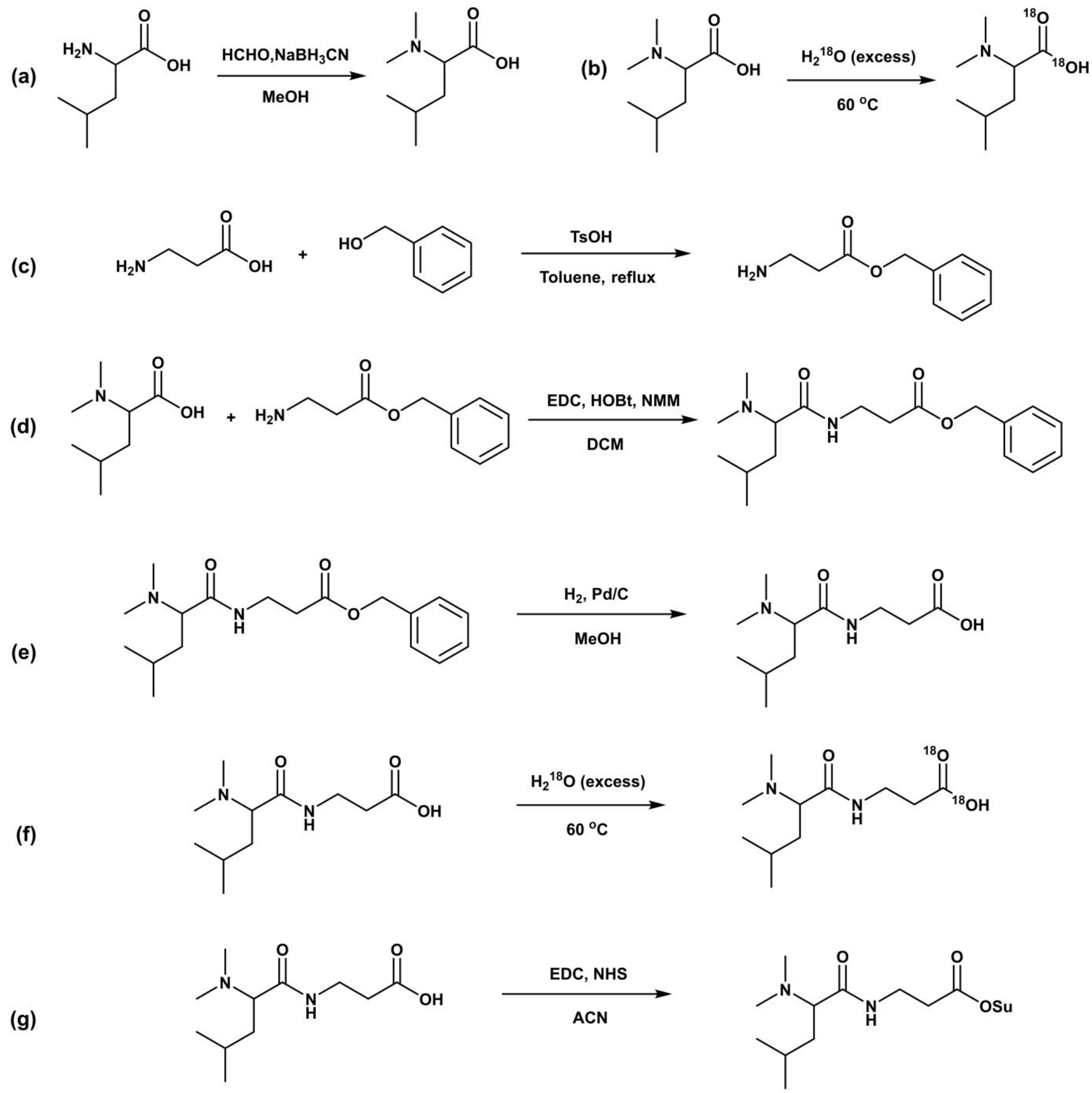

Scheme S1. Chemical synthetic route of the DiLeuC isobaric tag. Reaction steps (a–g) correspond to the labeled synthetic steps shown in the scheme.

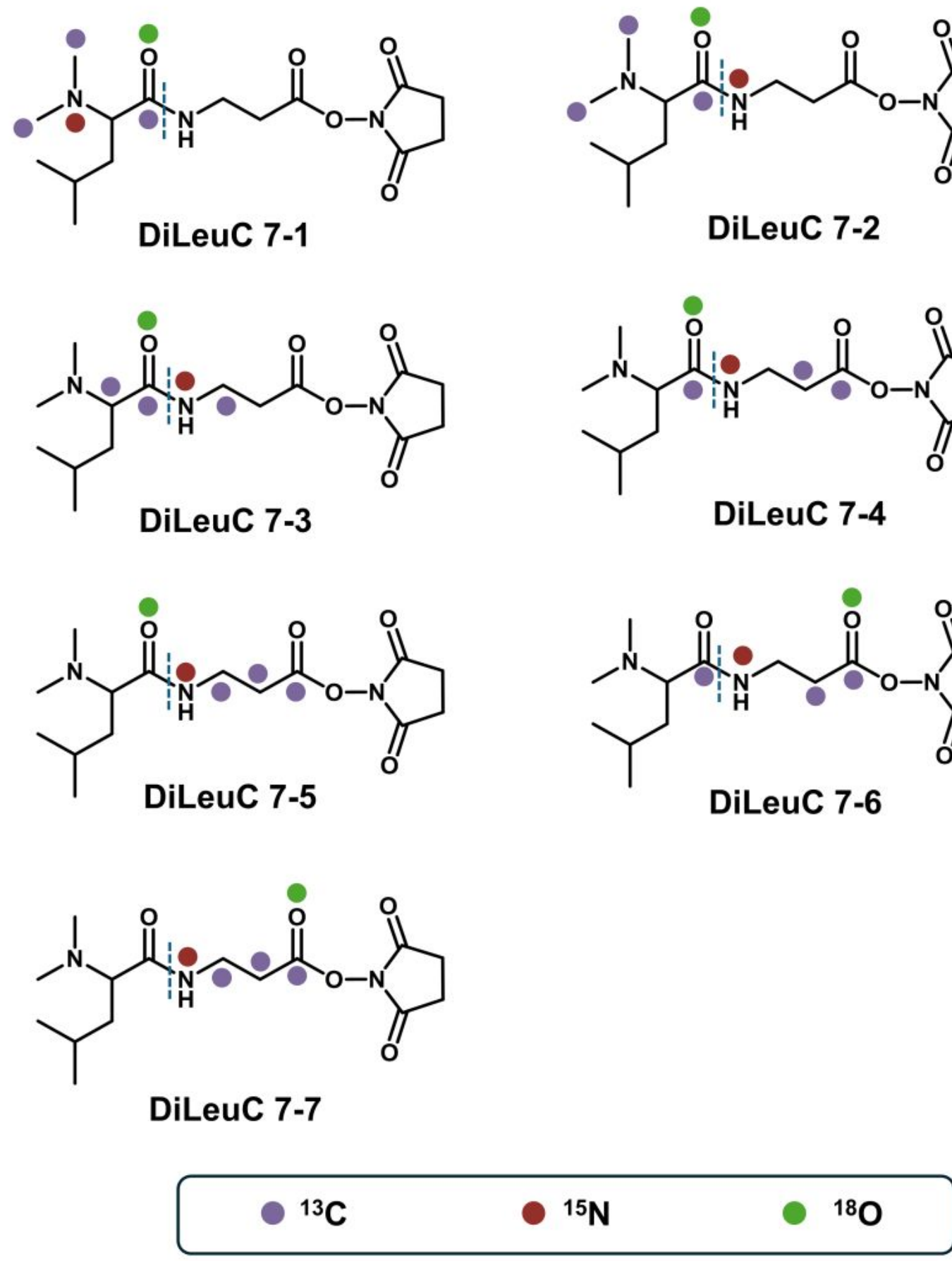

Figure S1. Isotopic distribution of 7-plex DiLeuC isobaric tag.

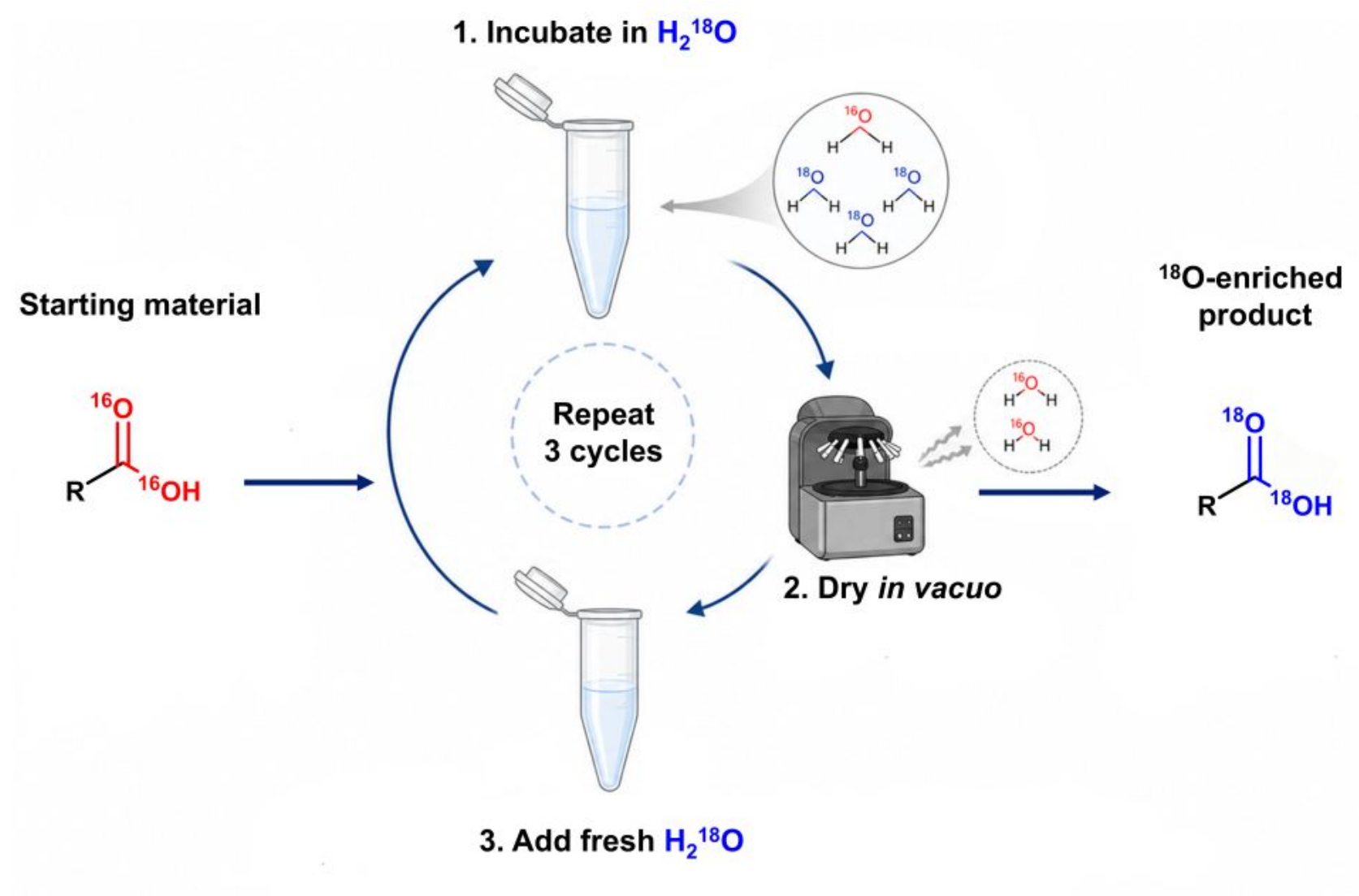

Figure S2. Iterative ^{18}O-exchange workflow. The carboxylic acid intermediate was incubated in $H_2{}^{18}O$ to exchange, dried in vacuo to remove $H_2{}^{16}O$ generated, and then reconstituted with fresh $H_2{}^{18}O$. The cycle was repeated three times to promote the formation of the ^{18}O-enriched product.

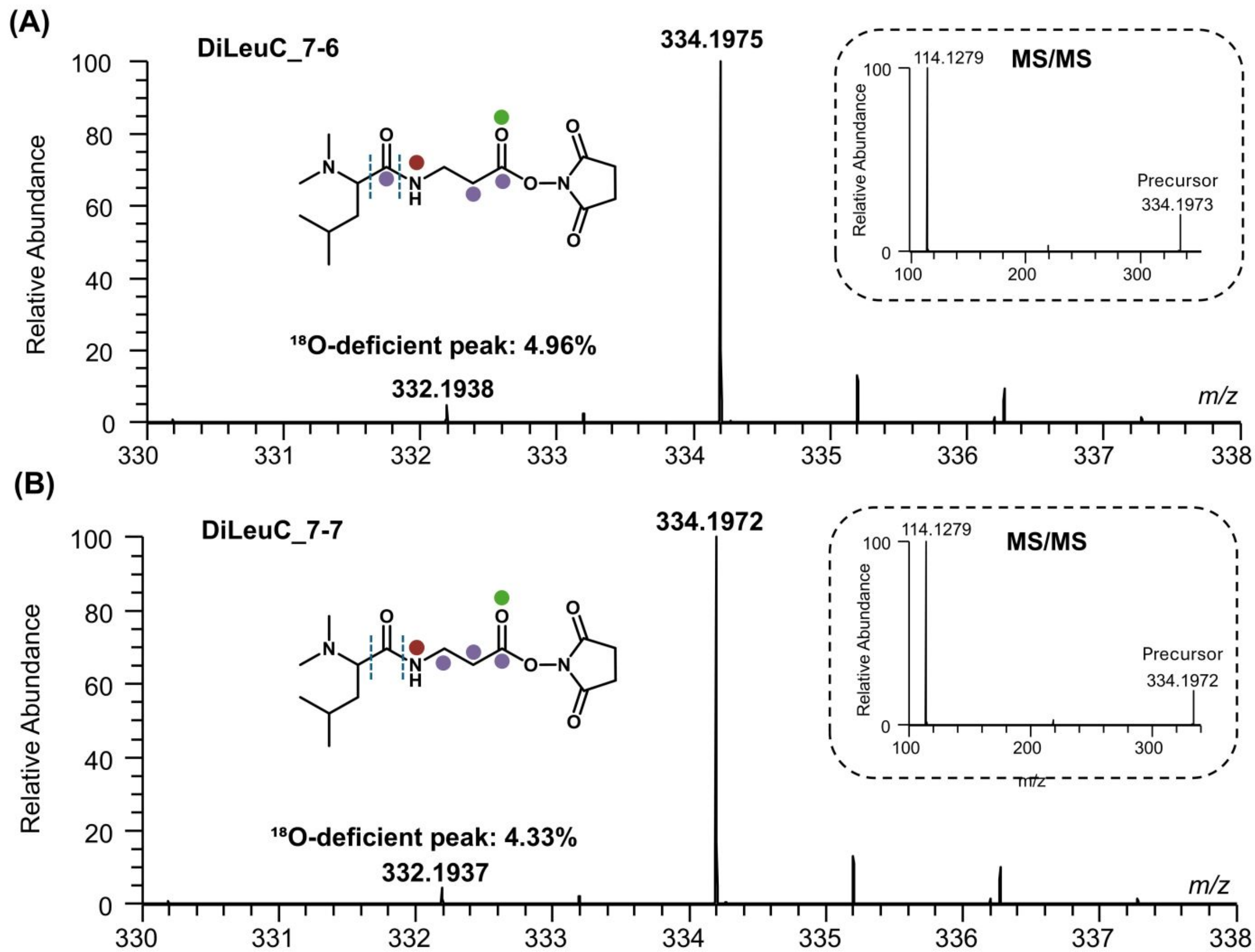

Figure S3. High-resolution MS characterization of optimized DiLeuC 7-6 and 7-7 reagents. HRMS spectra of purified DiLeuC_7-6 (A) and DiLeuC_7-7 (B) showed markedly reduced ^{18}O-deficient peaks at *m/z* 332.1938 and 332.1937, corresponding to 4.96% and 4.33% of the main peak. The inset MS/MS spectra showed characteristic DiLeuC fragmentation that generated reporter ion, confirming successful reagent synthesis.

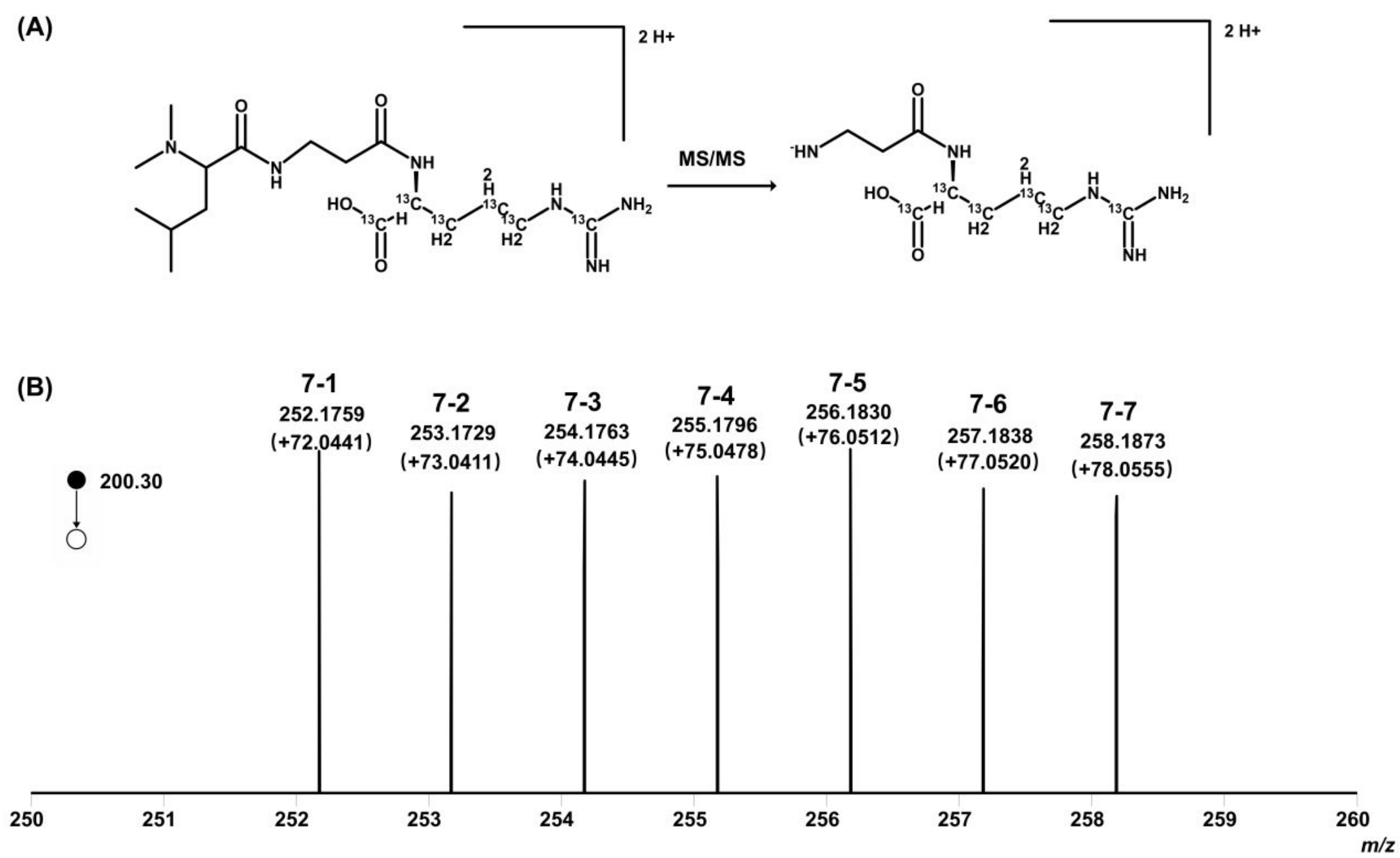

Figure S4. (A) MS/MS fragmentation scheme of DiLeuC-labeled $^{13}C_6$-arginine to generate the corresponding complementary ion. (B) MS/MS spectrum of seven complementary ions from seven plex DileuC-labeled $^{13}C_6$-arginine. The mass addition to the $^{13}C_6$-arginine (in Da) was marked in in parentheses.

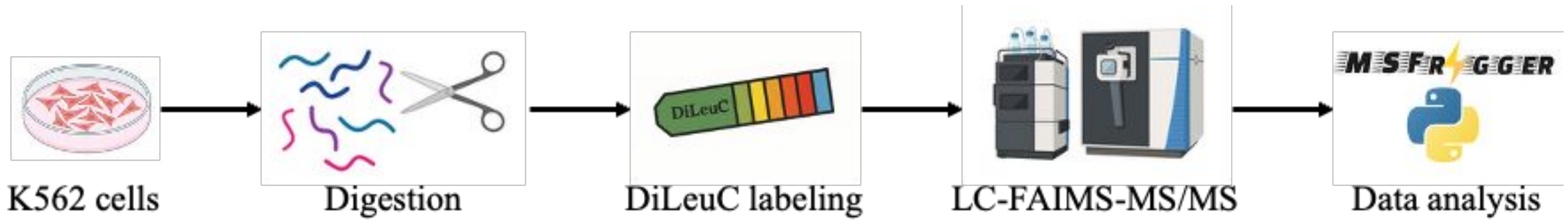

Figure S5. Workflow of the DiLeuC Strategy.

HCD 26%

(A)

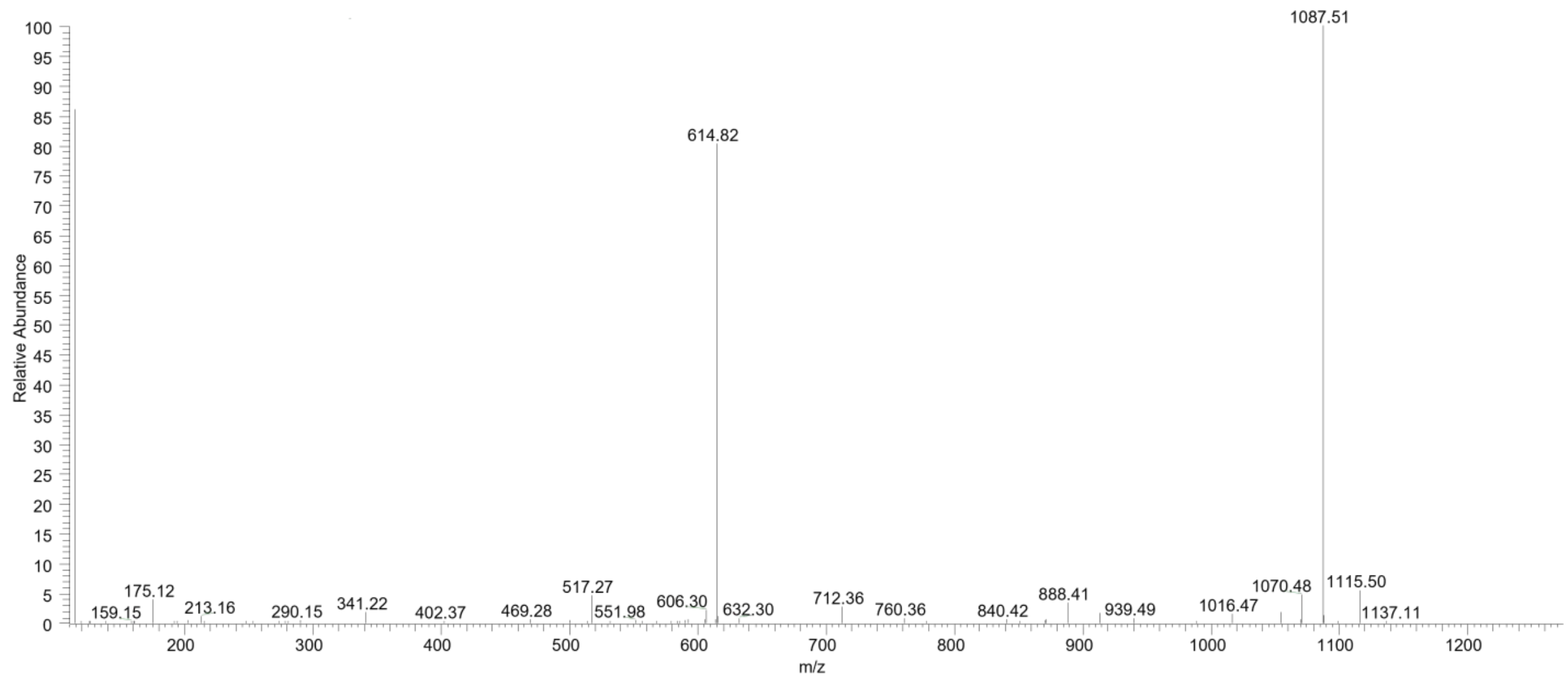

HCD 28%

(B)

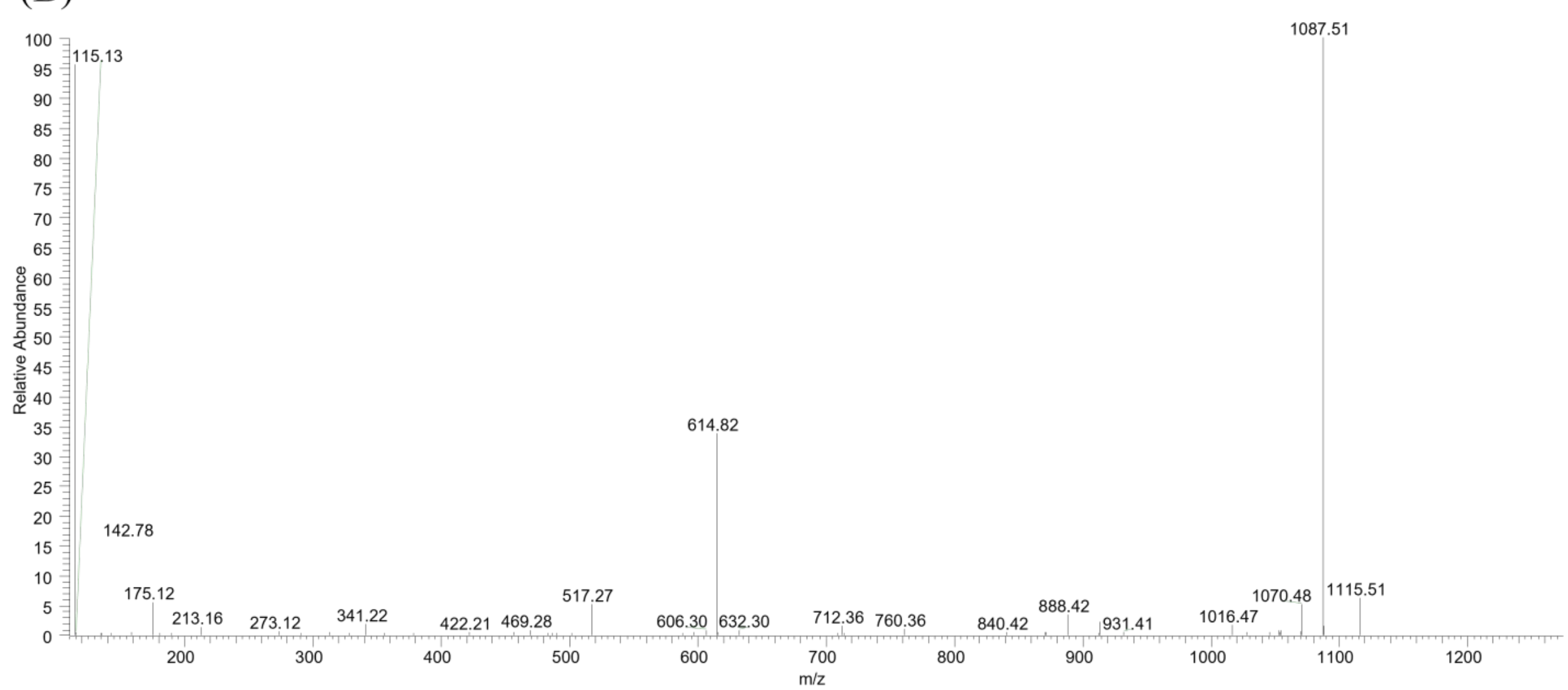

HCD 30%

(C)

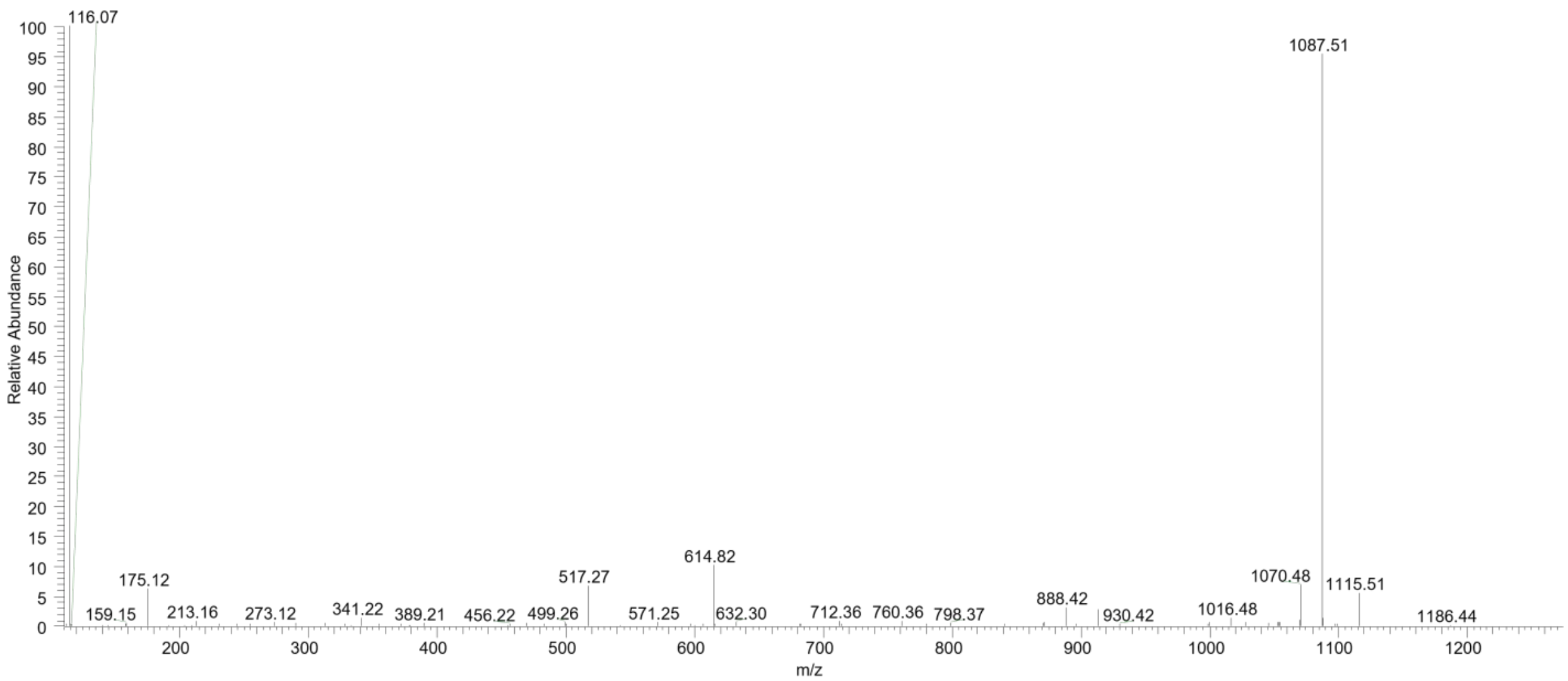

HCD 32%

(D)

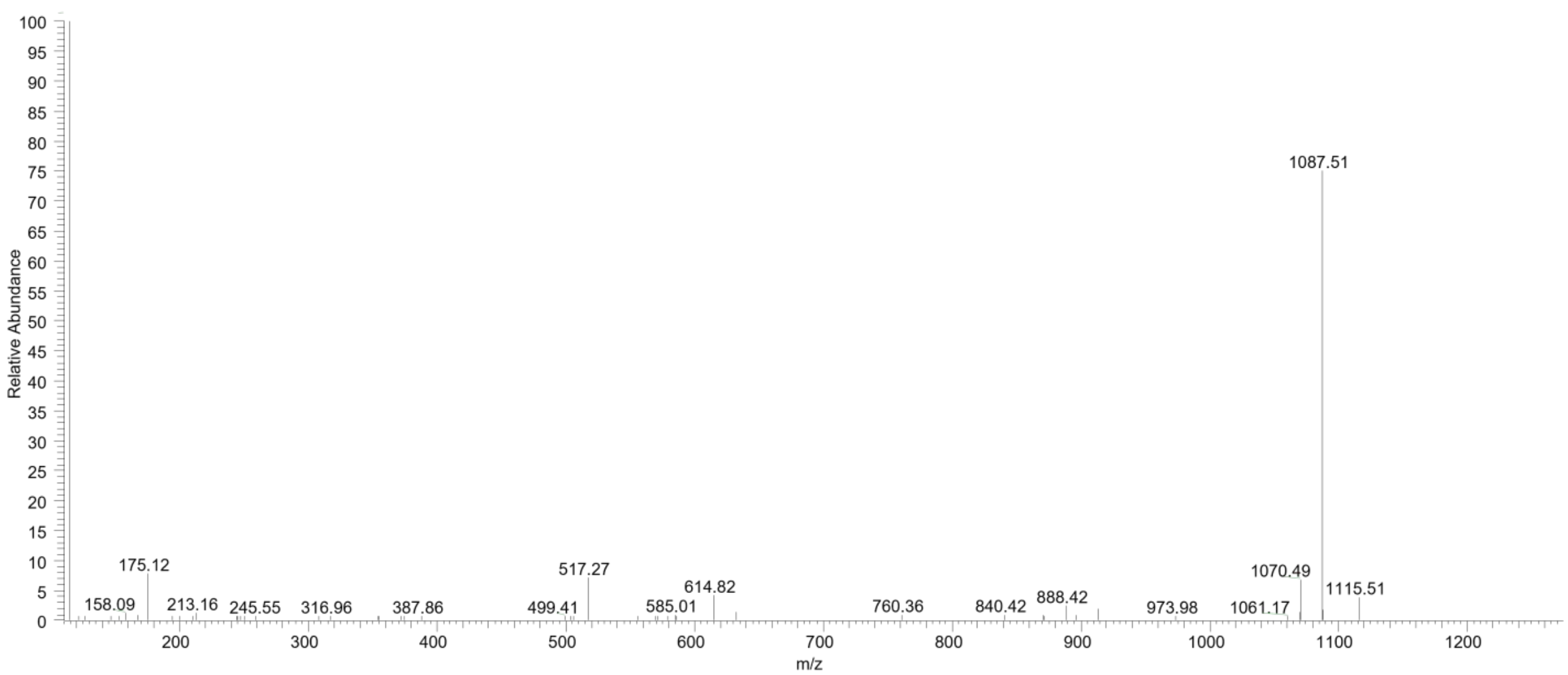

HCD 34%

(E)

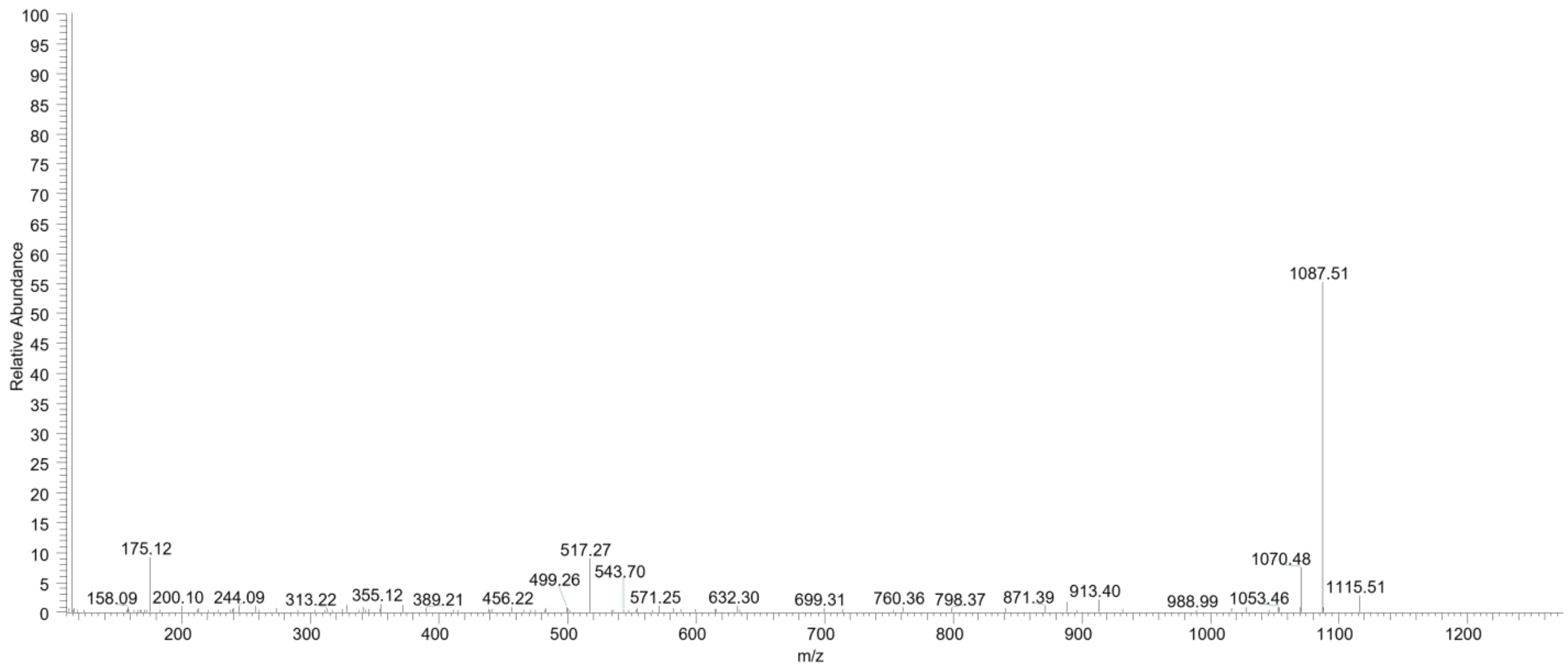

Figure S6. Representative MS/MS spectra illustrating DiLeuC complementary ion generation across different HCD collision energies. Representative MS/MS spectra of the same DiLeuC-labeled peptide acquired at NCE values of (A) 26%, (B) 28%, (C) 30%, (D) 32%, and (E) 34%.

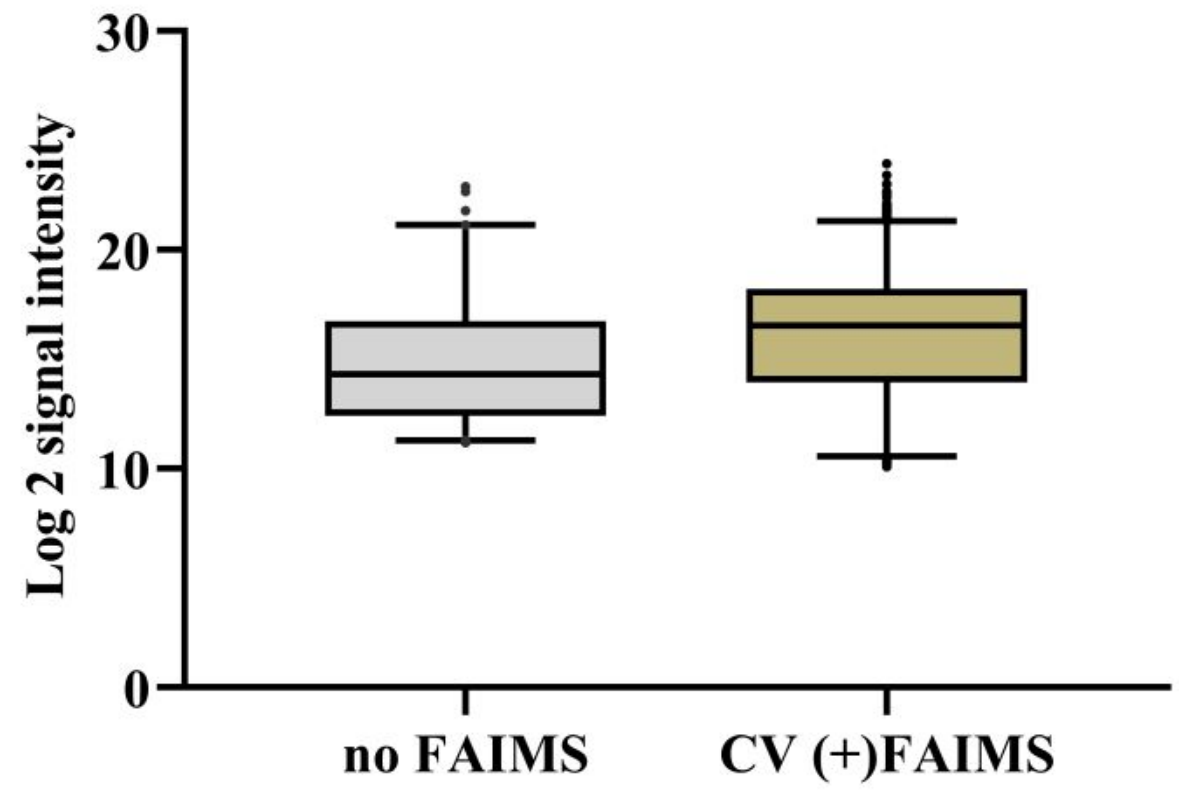

Figure S7. Comparison of signal intensity with and without FAIMS. Box plots showing the log2-transformed signal intensity distributions obtained without FAIMS and under the optimized FAIMS condition. The center line represents the median, the box represents the interquartile range, and the whiskers and individual points indicate the distribution and outliers, respectively.

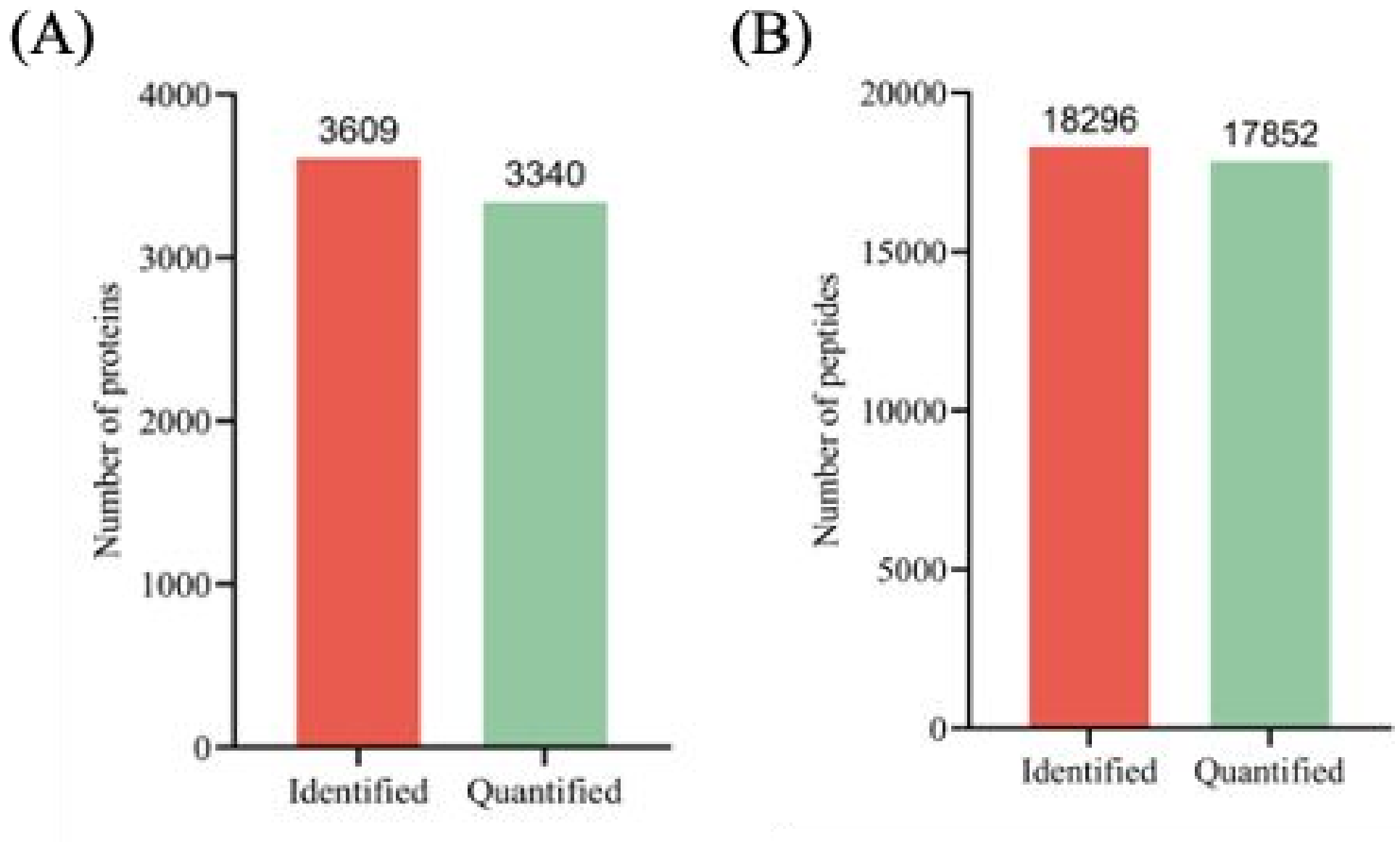

Figure S8. (A) Number of identified and quantified proteins in K562 cells. (B) Number of identified and quantified peptides in K562 cells.

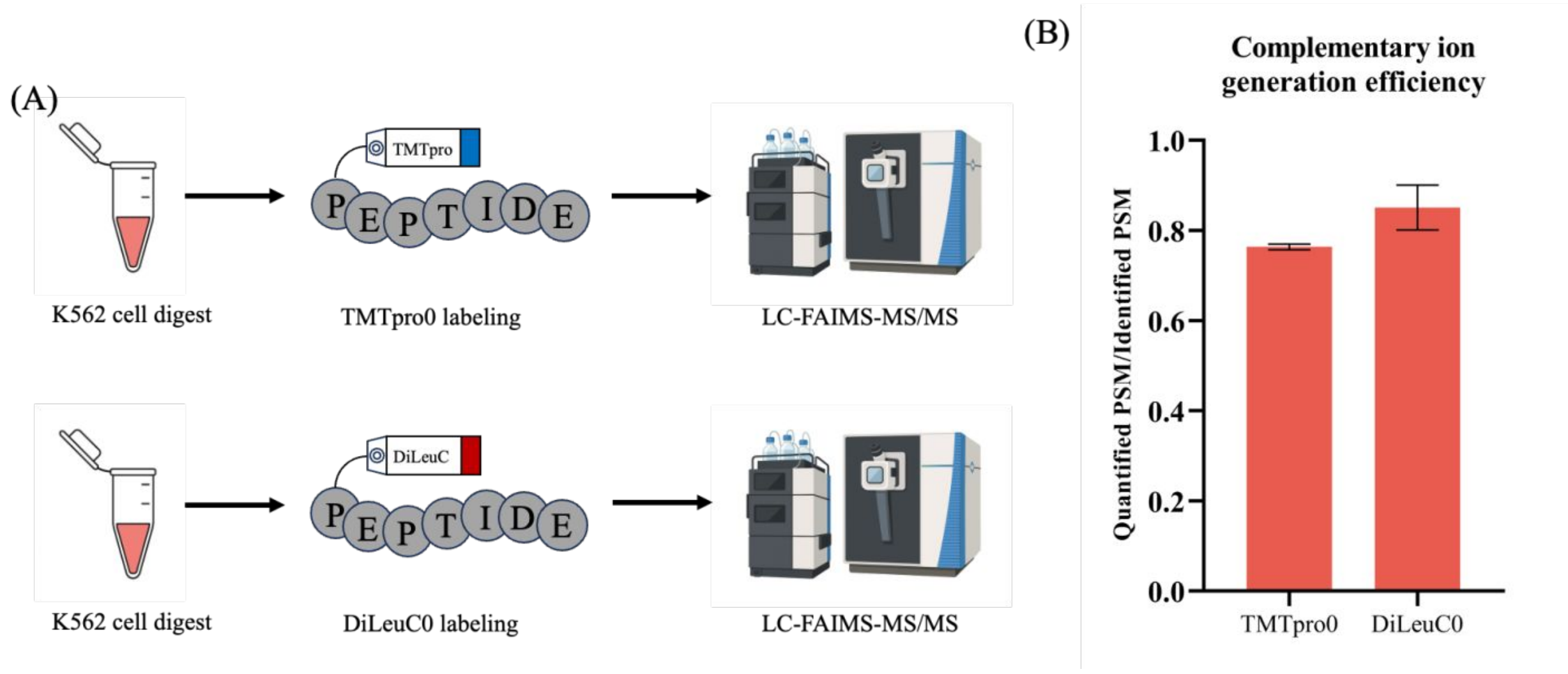

Figure S9. Comparison of complementary ion generation efficiency between DiLeuC zero and TMTpro zero methods. (A) Schematic workflow illustrating tryptic peptide labeling using DiLeuC zero and TMTpro zero reagents. (B) Bar plot showing the ratio of quantified PSMs to identified PSMs for both methods.

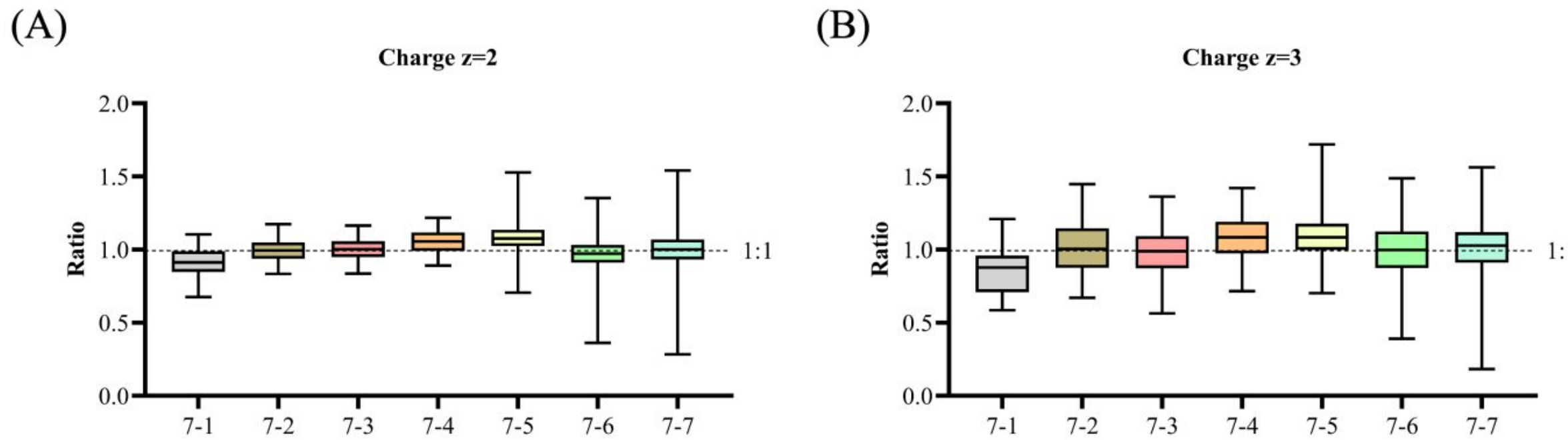

Figure S10. Charge-state-specific quantitative performance of 7-plex DiLeuC. Peptide-level quantitative distributions for the 1:1:1:1:1:1:1 PANC-1 sample analyzed separately for (A) doubly charged (z = 2) and (B) triply charged (z = 3) ions. The dashed line indicates the theoretical ratio of 1:1.

Table S1. Identification and complementary ion quantification performance under different FAIMS conditions.

FAIMS condition	Identified PSMs	PSMs with quantifiable complementary ions	Quantified PSMs/identified PSMs
-35V	129	57	0.44
-45V	843	457	0.54
-55V	1766	1248	0.71
-65V	1464	1082	0.74
-75V	704	487	0.69
-35-55V	1348	1000	0.74
-45-65V	3483	2840	0.82
-55-75V	1714	1312	0.77
-30-55-70V	1493	1210	0.81

Table S2. Comparison of quantitative accuracy and precision reported for complementary ion– and reporter ion–based isobaric quantification strategies.

Method	Plex	Instrument	Sample type and Benchmark design	Accuracy : median measured (deviation from theoretical ratio)	Precision (median CV)	Reference
DiLeuC (this work)	7	Exploris 480	PANC-1, 1:1:1:1:1:1:1	—	**13%** peptide level	This work
DiLeuC (this work)	7	Exploris 480	PANC-1, 1:2:5:10:5:2:1	—	**20%** peptide level	This work
DiLeuC (this work)	7	Exploris 480	K562 + yeast 5:1, yeast 1:2:5:10:5:2:1, K562 1:1:1:1:1:1:1	0.93 (-7%), 1.89 (-6%),4.57 (-9%), 9.48 (-5%), 5.27 (+5%),2.22 (+11%), 1.09 (+9%) ; peptide level	—	This work
DiLeuC,4-plex (previous work), complementary ion	4	Fusion Lumos	MCF7 + yeast 5:1, yeast 1:2:5:10, MCF7 1:1:1:1	2:1 to 2.02 (+1%); 5:1 to 5.15 (+3%); 10:1 to 9.57 (-4%); peptide level	not reported	Li et al.[2]
DiLeuC,4-plex (previous work),SPS-MS3, reporter ion	4	Fusion Lumos	MCF7 + yeast 5:1, yeast 1:2:5:10, MCF7 1:1:1:1	2:1 → 1.75 (-13%); 5:1 → 4.98 (-0.4%); 10:1 → 9.58 (-4%) ;peptide level	not reported	Li et al.[2]
DiLeuC,4-plex (previous work),MS2, reporter ion	4	Fusion Lumos	MCF7 + yeast 5:1, yeast 1:2:5:10, MCF7 1:1:1:1	2:1 → 1.04 (-48%); 5:1 → 3.31 (-34%); 10:1 → 5.11 (-49%); peptide level	not reported	Li et al.[2]

EASI-tag	6	Q Exactive HF-X	HeLa + yeast, yeast 1:3:10:10:3:1; HeLa 1:1:1:1:1:1	Medians not reported; ratio distributions centered on expected values; peptide level	yeast: **2.9%** (1:1), **7.2%** (1:3), **16.0%** (1:10), **17.0%** (1:10), **7.6%** (1:3); HeLa (1:1): 4.2 / 6.7 / 7.6 / 8.2 / 6.2% ; protein level	Winter et al.[3]
TMTc, complementary ion (full isotope envelope isolation)	5	Fusion Lumos	HeLa 1:1:1:1:1	not reported	**16%**, peptide level	Sonnett et al.[4]
TMTc+, complementary ion (1.0 Th isolation window)	5	Fusion Lumos	HeLa 1:1:1:1:1	not reported	**8%**, peptide level	Sonnett et al. [4]
TMTc+, complementary ion (0.5 Th isolation window)	5	Fusion Lumos	HeLa 1:1:1:1:1	not reported	**6%**, peptide level	Sonnett et al. [4]
TMTc+, complementary ion (0.4 Th isolation window)	5	Fusion Lumos	HeLa 1:1:1:1:1	not reported	**4%,** peptide level	Sonnett et al. [4]
TMTc+, complementary ion (0.4 Th isolation window)	5	Fusion Lumos	HeLa + yeast, yeast 1:10; HeLa 1:1	10:1 → 9.83 (-2%) , peptide level	not reported	Sonnett et al. [4]
TMT-MS3, reporter ion	5	Fusion Lumos	HeLa + yeast, yeast 1:10; HeLa 1:1	10:1 → 9.28 (-7%) , peptide level	not reported	Sonnett et al. [4]
TMT-MS2, reporter ion	5	Fusion Lumos	HeLa + yeast, yeast 1:10; HeLa 1:1	10:1 → 6.88 (-31%), peptide level	not reported	Sonnett et al. [4]

TMTpro-MS2, reporter ion	8	Fusion Lumos	HeLa : yeast 10:1, yeast 8 channels 0:1:5:10:10:5:1:0	5:1 → 2.6 (-48%); 10:1 → 4.6 (-54%) ;peptide level	not reported	Johnson et al.[5]
TMTpro-MS2 and FAIMS, reporter ion	8	Fusion Lumos	HeLa : yeast 10:1, yeast 8 channels 0:1:5:10:10:5:1:0	5:1 → 3.1 (-38%); 10:1 → 5.9 (-41%) ;peptide level	not reported	Johnson et al.[5]
TMTproC, complementary ion	8	Eclipse	HeLa : yeast 10:1, yeast 8 channels 0:1:5:10:10:5:1:0	5:1 → 4.3 (-14%); 10:1 → 8.3 (-17%); peptide level	not reported	Johnson et al.[5]
TMTpro, RTS-SPS-MS3	8	Eclipse	HeLa : yeast 10:1, yeast 8 channels 0:1:5:10:10:5:1:0	5:1 → 3.8 (-24%); 10:1 → 7.5 (-25%); peptide level	not reported	Johnson et al.[5]

Reference:

(1) Frost, D. C.; Greer, T.; Li, L. High-Resolution Enabled 12-Plex DiLeu Isobaric Tags for Quantitative Proteomics. *Anal Chem* **2015**, *87* (3), 1646–1654. https://doi.org/10.1021/ac503276z.

(2) Li, M.; Ma, M.; Li, L. Development of Novel Isobaric Tags Enables Accurate and Sensitive Multiplexed Proteomics Using Complementary Ions. *Anal. Bioanal. Chem.* **2023**, *415* (28), 6951–6960. https://doi.org/10.1007/s00216-023-04877-3.

(3) Winter, S. V.; Meier, F.; Wichmann, C.; Cox, J.; Mann, M.; Meissner, F. EASI-Tag Enables Accurate Multiplexed and Interference-Free MS2-Based Proteome Quantification. *Nat. Methods* **2018**, *15* (7), 527–530. https://doi.org/10.1038/s41592-018-0037-8.

(4) Sonnett, M.; Yeung, E.; Wühr, M. Accurate, Sensitive, and Precise Multiplexed Proteomics Using the Complement Reporter Ion Cluster. *Analytical Chemistry* **2018**, *90* (8), 5032–5039. https://doi.org/10.1021/acs.analchem.7b04713.

(5) Johnson, A.; Stadlmeier, M.; Wühr, M. TMTpro Complementary Ion Quantification Increases Plexing and Sensitivity for Accurate Multiplexed Proteomics at the MS2 Level. *Journal of Proteome Research* **2021**, *20* (6), 3043–3052. https://doi.org/10.1021/acs.jproteome.0c00813.
