# Supplementary material for: Accurate and Sensitive Multiplexed Proteomics Using the 7‑Plex DiLeuC Complementary Ion Strategy
Source: J Am Soc Mass Spectrom. 2026 Oct 6;37(11):2759–67. doi: 10.1021/jasms.6c00234 (PMC13741911; doi:10.1021/jasms.6c00234)
Supplement: Supplementary file 2 [file js6c00234_si_002.pdf]

```
import os
import pandas as pd
from pyteomics import mzml
import numpy as np

DEFAULT_PPM_TOLERANCE = 20
DEFAULT_INTENSITY_THRESHOLD = 0

def load_fragpipe_psm(file_path):
    df = pd.read_csv(file_path, sep='\t')
    df.columns = df.columns.str.strip()

    if 'Observed M/Z' not in df.columns and 'Calibrated Observed M/Z' not in df.columns:
        raise KeyError("The input file must contain either 'Observed M/Z' or 'Calibrated Observed M/Z'.")

    peptides = []
    for _, row in df.iterrows():
        peptide_info = row.to_dict()
        precursor_mz = row['Observed M/Z'] if 'Observed M/Z' in df.columns else row['Calibrated Observed M/Z']

        if row['Charge'] == 1:
            new_charges = [2, 3]
            for new_charge in new_charges:
                new_info = peptide_info.copy()
                new_info['precursor_mz'] = (precursor_mz + (new_charge - 1) *
```

```
1.00783) / new_charge
                new_info['Charge'] = new_charge
                new_info['conversion_flag'] = True
                peptides.append(new_info)
        else:
            peptide_info['precursor_mz'] = precursor_mz
            peptide_info['Charge'] = row['Charge']
            peptide_info['conversion_flag'] = False
            peptides.append(peptide_info)

    return peptides


def calculate_ppm_tolerance(mz, ppm_tolerance):
    return mz * ppm_tolerance / 1e6


def extract_complementary_ion_intensity(peptides, dia_file_path,
ppm_tolerance=DEFAULT_PPM_TOLERANCE,
intensity_threshold=DEFAULT_INTENSITY_THRESHOLD):
    complementary_ion_intensities = []
    pre_unique_modified_peptides = set()
    pre_unique_proteins = set()
    post_unique_modified_peptides = set()
    post_unique_proteins = set()

    for peptide_info in peptides:
        pre_unique_modified_peptides.add(peptide_info.get('Modified Peptide', ''))
        pre_unique_proteins.add(peptide_info.get('Protein', ''))
```

```
    spectra = mzml.read(dia_file_path)
    spectrum_dict = {}
    for spectrum in spectra:
        if spectrum['ms level'] == 2:
            spectrum_id_str = spectrum['id']
            if 'scan=' in spectrum_id_str:
                scan_num = int(spectrum_id_str.split('scan=')[-1])
                spectrum_dict[scan_num] = spectrum

    for peptide_info in peptides:
        precursor_mz = peptide_info['precursor_mz']
        charge = peptide_info['Charge']
        spectrum_id = peptide_info['Spectrum']

        if charge - 1 == 0:
            continue

        complementary_ion_mz = (precursor_mz * charge - 142.1138) / (charge - 1)
        tolerance_12C = calculate_ppm_tolerance(complementary_ion_mz,
ppm_tolerance)

        try:
            spectrum_id_int = int(spectrum_id.split('.')[-3])
        except ValueError:
            continue
```

```
            if spectrum_id_int in spectrum_dict:
                spectrum = spectrum_dict[spectrum_id_int]
                mz_array = spectrum['m/z array']
                intensity_array = spectrum['intensity array']

                idx_12C = np.where(np.abs(mz_array - complementary_ion_mz) <=
tolerance_12C)[0]

                if len(idx_12C) > 0:
                    max_intensity_idx_12C =
idx_12C[np.argmax(intensity_array[idx_12C])]
                    intensity_12C = intensity_array[max_intensity_idx_12C]

                    if intensity_12C >= intensity_threshold:
                        matched_mz_12C = mz_array[max_intensity_idx_12C]
                        new_row = peptide_info.copy()
                        new_row.update({
                            'complementary_ion_mz': matched_mz_12C,
                            'complementary_ion_intensity': intensity_12C,
                        })
                        complementary_ion_intensities.append(new_row)

post_unique_modified_peptides.add(peptide_info.get('Modified Peptide', ''))
                        post_unique_proteins.add(peptide_info.get('Protein', ''))

    result_df = pd.DataFrame(complementary_ion_intensities)

    stats = {
```

```
        'psms_before': len(peptides),
        'unique_peptides_before': len(pre_unique_modified_peptides),
        'unique_proteins_before': len(pre_unique_proteins),
        'psms_after': len(complementary_ion_intensities),
        'unique_peptides_after': len(post_unique_modified_peptides),
        'unique_proteins_after': len(post_unique_proteins)
    }

    return result_df, stats


# ------------------------- Batch processing -------------------------

def process_folder(input_folder, output_folder,
ppm_tolerance=DEFAULT_PPM_TOLERANCE,
intensity_threshold=DEFAULT_INTENSITY_THRESHOLD):
    """
    Batch-process matched FragPipe TSV and mzML files in a folder.

    Parameters
    ----------
    input_folder : str or pathlib.Path
        Folder containing FragPipe .tsv files and corresponding .mzML files.
        Each TSV file must have a matching mzML file with the same base name.
    output_folder : str or pathlib.Path
        Folder where complementary-ion result CSV files and summary.csv
        will be written.
```

```
    ppm_tolerance : float
        Mass tolerance in ppm for complementary-ion matching.
    intensity_threshold : float
        Minimum complementary-ion intensity required for quantification.
    """
    input_folder = Path(input_folder)
    output_folder = Path(output_folder)
    output_folder.mkdir(parents=True, exist_ok=True)

    tsv_files = sorted(input_folder.glob("*.tsv"))
    summary_stats_list = []

    for tsv_path in tsv_files:
        base_name = tsv_path.stem
        mzml_path = input_folder / f"{base_name}.mzML"

        if not mzml_path.exists():
            print(f"Warning: {mzml_path.name} not found for {tsv_path.name};
skipping.")
            continue

        print(f"\nProcessing {tsv_path.name} and {mzml_path.name}...")

        peptides = load_fragpipe_psm(tsv_path)
        complementary_ion_data, stats = extract_complementary_ion_intensity(
            peptides,
            mzml_path,
```

```
                ppm_tolerance=ppm_tolerance,
                intensity_threshold=intensity_threshold,
            )

            output_csv_path = output_folder /
f"{base_name}_complementary_ions.csv"
            complementary_ion_data.to_csv(output_csv_path, index=False)

            stats_row = {
                "Sample": base_name,
                "PSMs_before": stats["psms_before"],
                "Unique_modified_peptides_before": stats["unique_peptides_before"],
                "Unique_proteins_before": stats["unique_proteins_before"],
                "PSMs_after": stats["psms_after"],
                "Unique_modified_peptides_after": stats["unique_peptides_after"],
                "Unique_proteins_after": stats["unique_proteins_after"],
            }
            summary_stats_list.append(stats_row)

        summary_df = pd.DataFrame(summary_stats_list)
        summary_csv_path = output_folder / "summary.csv"
        summary_df.to_csv(summary_csv_path, index=False)

        print(f"\nAll files processed. Summary saved to: {summary_csv_path}")


if __name__ == "__main__":
```

```
import argparse


parser = argparse.ArgumentParser(
    description=(
        "Extract DiLeuC complementary-ion intensities from FragPipe PSM
TSV "
        "files and corresponding mzML files."
    )
)
parser.add_argument(
    "-i",
    "--input",
    required=True,
    help="Input folder containing matched .tsv and .mzML files.",
)
parser.add_argument(
    "-o",
    "--output",
    required=True,
    help="Output folder for complementary-ion CSV files and summary.csv.",
)
parser.add_argument(
    "--ppm",
    type=float,
    default=DEFAULT_PPM_TOLERANCE,
    help=f"Mass tolerance in ppm (default:
{DEFAULT_PPM_TOLERANCE}).",
```

```
)
parser.add_argument(
    "--intensity-threshold",
    type=float,
    default=DEFAULT_INTENSITY_THRESHOLD,
    help=(
        "Minimum complementary-ion intensity required for quantification "
        f"(default: {DEFAULT_INTENSITY_THRESHOLD})."
    ),
)

args = parser.parse_args()

process_folder(
    args.input,
    args.output,
    ppm_tolerance=args.ppm,
    intensity_threshold=args.intensity_threshold,
)
```
